# Cisplatin resistance in non-small cell lung cancer cells is associated with an abrogation of cisplatin-induced G_2_/M cell cycle arrest

**DOI:** 10.1371/journal.pone.0181081

**Published:** 2017-07-26

**Authors:** Navin Sarin, Florian Engel, Ganna V. Kalayda, Mareike Mannewitz, Jindrich Cinatl, Florian Rothweiler, Martin Michaelis, Hisham Saafan, Christoph A. Ritter, Ulrich Jaehde, Roland Frötschl

**Affiliations:** 1 Institute of Pharmacy, Clinical Pharmacy, University of Bonn, Bonn, Germany; 2 Federal Institute for Drugs and Medical Devices (BfArM), Bonn, Germany; 3 Institute of Medical Virology, Goethe University Hospital Frankfurt, Frankfurt/Main, Germany; 4 Centre for Molecular Processing and School of Biosciences, University of Kent, Canterbury United Kingdom; 5 Institute of Pharmacy, Clinical Pharmacy, University of Greifswald, Greifswald, Germany; University of South Alabama Mitchell Cancer Institute, UNITED STATES

## Abstract

The efficacy of cisplatin-based chemotherapy in cancer is limited by the occurrence of innate and acquired drug resistance. In order to better understand the mechanisms underlying acquired cisplatin resistance, we have compared the adenocarcinoma-derived non-small cell lung cancer (NSCLC) cell line A549 and its cisplatin-resistant sub-line A549^r^CDDP^2000^ with regard to cisplatin resistance mechanisms including cellular platinum accumulation, DNA-adduct formation, cell cycle alterations, apoptosis induction and activation of key players of DNA damage response. In A549^r^CDDP^2000^ cells, a cisplatin-induced G_2_/M cell cycle arrest was lacking and apoptosis was reduced compared to A549 cells, although equitoxic cisplatin concentrations resulted in comparable platinum-DNA adduct levels. These differences were accompanied by changes in the expression of proteins involved in DNA damage response. In A549 cells, cisplatin exposure led to a significantly higher expression of genes coding for proteins mediating G_2_/M arrest and apoptosis (mouse double minute 2 homolog (MDM2), xeroderma pigmentosum complementation group C (XPC), stress inducible protein (SIP) and p21) compared to resistant cells. This was underlined by significantly higher protein levels of phosphorylated Ataxia telangiectasia mutated (pAtm) and p53 in A549 cells compared to their respective untreated control. The results were compiled in a preliminary model of resistance-associated signaling alterations. In conclusion, these findings suggest that acquired resistance of NSCLC cells against cisplatin is the consequence of altered signaling leading to reduced G_2_/M cell cycle arrest and apoptosis.

## Introduction

Non-small cell lung cancer (NSCLC) is associated with high incidence and mortality [1]. Cisplatin (cis-diamminedichloroplatinum (II)) is one of the common constituents of first-line treatment after surgery [2]. The absolute effect of cisplatin chemotherapy after five years was shown to be a decrease of lung cancer associated death by 6.9% compared to an untreated control group [3]. The precise antineoplastic mechanism of cisplatin remains not completely understood [4]. Cisplatin cytotoxicity has been suggested to be predominantly mediated by nuclear DNA binding, mainly via intrastrand DNA crosslinks leading to cell cycle arrest and subsequent apoptosis [5]. However, this view may be an oversimplification since only around 1% of the intracellular cisplatin binds to nuclear DNA. Cisplatin becomes intracellularly activated into an aquatic complex that reacts with numerous membrane and cytoplasmic components, suggesting that the drug may also exert cytotoxic effects by mechanisms independent of nuclear DNA binding [6].

The efficacy of cisplatin-based chemotherapies is limited by the occurrence of innate and acquired drug resistance. The mechanisms underlying cisplatin resistance are complex indicating that there is no single mechanism-based strategy to overcome cisplatin resistance [4,7,8]. Galuzzi et al. classified the cisplatin resistance mechanisms into four categories: (i) pre-target resistance, preventing the binding of cisplatin to DNA by reduced cellular accumulation or enhanced binding to cytoplasmic components; (ii) on-target resistance, by tolerating or repairing cisplatin-DNA adducts; (iii) post-target resistance, by several alterations or defects in signaling pathways in response to DNA damage by cisplatin; and (iv) off-target resistance, by mechanisms, which do not directly involve cisplatin-initiated signals but enable cells to circumvent cisplatin-induced cell death [4]. Various proteins, genes or pathways were found to be involved in resistance against cisplatin [7,8], but any of the mechanisms was identified as the crucial one. Therefore, a single mechanism-based strategy to overcome cisplatin resistance will unlikely be successful. Consequently, the knowledge of extensive cytoplasmic and nuclear interactions of cisplatin and the multifactorial nature of resistance has driven us towards a more systematic approach, leading in the far future to a holistic model to explain the development of cisplatin resistance in NSCLC cell. Hence, we chose a bottom-up approach to compare cisplatin-sensitive and -resistant NSCLC cells to first outline the differences in biomolecular actions upon cisplatin exposure and then unveil the underlying mechanisms responsible.

## Materials and methods

### Drugs

*Cis*-diamminedichloroplatinum (II) (cisplatin) was obtained from Sigma–Aldrich, Steinheim, Germany and dissolved in 0.9% NaCl at a final concentration of 1.5 g/L. The platinum concentration in the stock solution was verified by flameless atomic absorption spectroscopy (AAS). Aliquots were stored at -20°C and thawed immediately before use.

### Cell lines

The human non-small cell lung carcinoma (NSCLC) cell line A549 was obtained from ATCC (Manassas, VA, USA) and its cisplatin resistant sub-line A549^r^CDDP^2000^ was derived from the Resistant Cancer Cell Line (RCCL) collection (http://www.kent.ac.uk/stms/cmp/RCCL/RCCLabout.html). The sub-line had been established by adapting the growth of A549 cells in the presence of increasing concentrations of cisplatin until a final concentration of 2000 ng/mL cisplatin as described previously [9]. A549 cells were grown in IMDM (PAN-Biotech, Aidenbach, Germany) containing 4 mM L-glutamine, supplemented with 10% fetal calf serum, 100 I.E./mL penicillin and 0.1 mg/mL streptomycin. The medium of the A549^r^CDDP^2000^ cells additionally contained 2 μg/mL cisplatin. Cells were cultivated as monolayers in a humidified atmosphere at 37°C and 5% CO_2_. For all experiments, cells were allowed to attach overnight, experienced 4 h of serum starvation and were subsequently treated with cisplatin for 24 h in IMDM medium without any supplement. Prior serum starvation and the use of serum-free medium avoided the influence of growth factors in serum on cellular signaling. It however restricted the incubation time to 24h as longer treatment can lead to starvation effects and apoptosis [10]. The cisplatin concentrations used were cell line dependent and based on the respective EC_10_. Both cell lines were treated with 11 μM cisplatin (EC_10_ of sensitive cell line). The resistant sub-line was besides treated with 34 μM cisplatin (the respective EC_10_). In the following, equimolar treatment refers to treatment of sensitive and resistant cell line with 11 μM cisplatin and equitoxic treatment refers to treatment of sensitive cell line with 11 μM cisplatin and resistant cell line with 34 μM cisplatin.

### Cisplatin cytotoxicity

MTT assays were performed to determine cisplatin cytotoxicity via cancer cell viability in response to cisplatin. Cisplatin concentrations that resulted in 90% cell viability relative to untreated control (EC_10_) were determined. The assay was performed as previously described [11] with slight modification. Briefly, 8000 cells were seeded in a 96-well microtiter plate and kept at 37°C and 5% CO_2_ overnight. After 4 h of serum starvation, cells were treated with different concentrations of cisplatin in growth medium without any supplements for 24 h. Following incubation, cells were treated with 3-(4,5-dimethylthiazol-2-yl)-2,5-diphenyltetrazolium bromide (MTT, Sigma–Aldrich, Steinheim, Germany) solution [5 mg/mL dissolved in phosphate-buffered saline (PBS)] for 1 h. After dissolving the formazan crystals in DMSO, absorbance of the converted dye was measured at 595 nm with background subtraction at 690 nm using the Multiwell-Reader Multiskan EX^®^ (Thermo Fisher Scientific, Waltham, MA, USA). EC_50_ and EC_10_ values (drug concentrations that reduce cell viability by 50% or 10%, respectively) were calculated using the software Prism^TM^ (GraphPad Software, La Jolla, CA, USA).

### Cellular platinum accumulation

The cellular platinum accumulation was measured to evaluate differences in platinum uptake in the cell lines, which contributes to resistance development. 2.5 × 10^5^ sensitive cells and 5 × 10^5^ of the resistant variant per well were seeded into 6-well plates and kept at 37°C and 5% CO_2_ overnight. After 4 h of serum starvation and treatment with cisplatin for 24 h, cells were washed with ice-cold PBS, trypsinized and centrifuged for 4 minutes at 1500 x g. After lysing the cell pellet in 50 μL concentrated HNO_3_ at 80°C for 1 h, total platinum was measured by flameless atomic absorption spectrometry. The diluted sample was injected into a graphite tube and after vaporization and atomization, platinum absorption was measured at 265.9 nm and 2700°C. The cellular platinum content was determined in relation to cellular protein content, which was determined by the BCA assay (BCA protein assay kit, Merck KGaA, Darmstadt, Germany).

### Cisplatin-DNA adducts

Cisplatin-DNA adducts were measured by immunoblotting. After 4 h of serum starvation and treatment with different concentrations of cisplatin for 24 h, total DNA was isolated with the RNeasy^TM^ Kit (Qiagen, Venlo, Netherlands). 1 μg of DNA was dissolved in Tris/EDTA (TE) buffer and denatured at 95°C for 10 min. Subsequently the DNA was spotted on Hybond^TM^ nitrocellulose membranes (GE Healthcare, Chalfont, St Giles, United Kingdom) with a slot blot manifold (GE Healthcare, Chalfont, St Giles, United Kingdom) by a vacuum of 35 kPa (kilo-Pascal). After denaturation with 0.4 M NaOH for 45 min on a drenched filter paper the membranes were blocked with 5% (w/v) non- fat dry milk powder (Bio-Rad Laboratories, Hercules, CA, USA) in TBS (tris-buffered saline) with 0.1% (v/v) Tween-20 (≙ TBS-T, Bio-Rad Laboratories, Hercules, CA, USA) overnight at 4°C. Subsequently, the membranes were incubated with the antibody against cisplatin-DNA adducts (Merck Millipore, Billerica, MA, USA), diluted 1:1000 in TBS-T with 5% (w/v) non-fat dry milk powder, for 2 h at room temperature to detect 1,2-d(GG) DNA intrastrand cross links. The membranes were washed 3 times with TBS-T followed by incubation with the secondary horse radish phosphatase (HRP) coupled antibody (1:1000, Antibodies-online.com, Aachen, Germany). Antibody complexes were detected using the enhanced chemiluminescence (ECL) reagent (Thermo Fisher Scientific, Waltham, MA, USA) and densitometric analysis was carried out using the AIDA^TM^ 4 software (Raytest, Straubenhardt, Germany).

### Cell cycle

Briefly, 5 × 10^5^ sensitive cells and 1 × 10^6^ cells of the resistant sub-line were seeded into T25 flasks and kept at 37°C and 5% CO_2_ overnight. After 4 h of serum starvation and cisplatin treatment for 24 h, the supernatant was collected and cells were washed once with PBS, harvested with AccuMax^TM^ (GE Healthcare, Chalfont, St Giles, United Kingdom) and transferred to the consolidated supernatants. Subsequently, cells were centrifuged for 5 min at 200 x g, supernatant was discarded and cells were fixed with 79% ethanol for 24 h at 4°C. After fixation the cells were centrifuged for 5 min at 1400 x g, washed with PBS and incubated with 100 μg/mL RNase A (Life Technologies, Carlsbad, CA, USA) for 30 min at room temperature. After staining with 5 μL propidium iodide (0.1 mg/mL in PBS, Sigma–Aldrich, Steinheim, Germany) samples were analyzed using flow cytometry (FACSCalibur^TM^, BD Bioscience, Franklin Lakes, NJ, USA).

### Apoptosis assay

Apoptosis was investigated using the BD Pharmingen™ FITC Annexin V Apoptosis Detection Kit (BD Biosciences, Franklin Lakes, NJ, USA). Briefly, 2.5 × 10^5^ sensitive cells and 5 × 10^5^ cells of the resistant sub-line per well were seeded into 6-well plates and kept at 37°C and 5% CO_2_ overnight. After 4 h of serum starvation and 24 h treatment, trypsinized cells in growth medium were centrifuged for 4 min at 1500 × g. The supernatant was discarded and 500 μL binding buffer (BD Bioscience, Franklin Lakes, NJ, USA) was added to resuspend the pellet. 5 μL propidium iodide and 5 μL Annexin V-FITC were added to 100 μL of the cells in binding buffer. After 15 min of incubation on ice, samples were analyzed using flow cytometry (FACSCalibur^TM^, BD Bioscience, Franklin Lakes, NJ, USA).

### RNA isolation, cDNA synthesis and RT-PCR

RNA was isolated using the RNeasy^TM^ Mini Kit (Qiagen, Venlo, Netherlands) and quantified photometrically with a Nanodrop^TM^ 1000 (Thermo Fisher Scientific, Waltham, MA, USA). Subsequent cDNA synthesis was performed for 60 min at 42°C. Reaction mixture was composed of 2 μl water (Qiagen, Venlo, Netherlands), 1.5 μl 10 x buffer (Life Technologies, Carlsbad, CA, USA), 1.1 μl MgCl_2_ (25 mM; Life Technologies, Carlsbad, CA, USA), 1.5 μl dithiothreitol (100 mM; Life Technologies, Carlsbad, CA, USA), 1.5 μl dNTP (2,5 mM; Life Technologies, Carlsbad, CA, USA), 0.6 μl Rnasin^®^ (20 u/μl; Life Technologies, Carlsbad, CA, USA), 0,3 μl oligo-dt-primer (Life Technologies, Carlsbad, CA, USA) and murine leukemia virus reverse transcriptase (50 u/μl Life Technologies, Carlsbad, CA, USA). RT-PCR was then performed with Probes Master Light Cycler (LC) 480 (Hoffmann La Roche, Basel, Switzerland). Hybridization probes and primers were purchased from TIB MOLBIOL (Berlin, Germany) and the sequences of those probes and primers are presented in (Table 1).

**Table 1:** Primer and probe sequences for RT-PCR.

**Gene**	**p53**	**SIP**
Forward-Primer	gCTGCTCAgATAgCgATggTCT	CggTACCATTgggCCAACTA
Reverse-Primer	gTACAgTCAgAgCCAACCTCAg	gCTgAgAAACCAgTgCAAgTATCTA
LC640-Probe	TCTgTCATCCAAATACTCCACACgC-PH	CCACAAACATTTTATTCAgCCTCTgg-PH
FL-Probe	gCACCACCACACTATgTCgAAAAgT-FL	TggTTggAggAAgAACTgACTTCA-FL
Annealing [°C]	57	57
**Gene**	**Actin**	**GADD45A (growth arrest and DNA damage inducible 45A)**
Forward-Primer	AgCCTCgCCTTTgCCgA	AAgCTgCTCAACgTCgACC
Reverse-Primer	CTggTgCCTggggCg	CgTCACCAgCACgCAgT
LC640-Probe	CgACgACgAgCgCggCgATATC-PH	AgCCACATCTCTgTCgTCgTCCTCgT-PH
FL-Probe	TTgCACATgCCggAgCCgTTg-FL	CTggATCAgggTgAAgTggATCTgCA-FL
Annealing [°C]	61	58
**Gene**	**XPC**	**p21**
Forward-Primer	CgATggggATgACCTCAgg	gAggCCgggATgAgTTg
Reverse-Primer	TTTCTTCCTCTTCTTCATTgCTg	gAgTggTAgAAATCTgTCATgCTg
LC640-Probe	TgTgCCTTCTTgAggTCACTTgg-PH	gTCTTgTACCCTTgTgCCTCgCTC-PH
FL-Probe	CATggTAgCCCCTCTCTTCAgATg-FL	gAggAAgACCATgTggACCTgTCAC-FL
Annealing [°C]	57	58
**Gene**	**MDM2**	** **
Forward-Primer	CAgATgAATTATCTggTgAACgA	
Reverse-Primer	AAACTgAATCCTgATCCAACC	
LC-Probe	TgTTgTgAAAgAAgCAgTAgCAgTgA-PH	
FL-Probe	CTggCTCTgTgTgTAATAAgggAgAT-FL	

### SDS-PAGE/Western blot

Cellular proteins were extracted using Denaturizing Cell Extraction Buffer (Life Technologies, Carlsbad, CA, USA). Protein concentration was determined by the BCA (bicinchoninic acid) assay (Pierce^TM^ BCA Protein Assay Kit, Life Technologies, Carlsbad, CA, USA). Whole protein extracts were separated on a 4–12% Bis-Tris polyacrylamide gel according to the method of Lämmli [12] using MOPS electrophoresis buffer. Proteins were transferred to a PVDF membrane (Bio-Rad Laboratories, Hercules, CA, USA), which was blocked after protein transfer with 5% (w/v) not-fat dry milk powder in TBS-T (Tween-20, 0.1% (v/v)) for 1 h at room temperature. Subsequently, the membranes were incubated overnight at 4°C with primary antibodies (Table 2) and washed three times for 10 min with TBS-T, followed by incubation with the secondary HRP-conjugated antibody (Santa Cruz Biotechnology, Dallas, TX, USA) for 1 h at room temperature. Antibody complexes were detected using the ECL reagent (Thermo Fisher Scientific, Waltham, MA, USA) and densitometric analysis was carried out using AIDA^TM^ 4 Software (Raytest, Straubenhardt, Germany). Protein signals were normalized to the housekeeping proteins GAPDH or α-actin. Experiments showed reproducibly that α-actin is expressed twofold higher in sensitive cells than in the resistant cells. Because some proteins are not detectable in untreated cells and therefore a fold of control analysis is not possible the normalization on α-actin had to be modified to keep the densitometric analysis of sensitive and resistant cells comparable. Therefore those proteins were normalized to α-actin/2 in sensitive cells. Representative whole western blots can be found in Supporting Information S9–S17 Figs)

**Table 2:** Antibodies with their respective dilution used for SDS-PAGE.

Antibody	Manufacturer	Dilution
α-Actin	Santa Cruz Biotechnology	1:1000
GAPDH	GeneTex	1:20000
p53-HRP	Santa Cruz Biotechnology	1:200
p21	Santa Cruz Biotechnology	1:200
pAtm	Santa Cruz Biotechnology	1:200
GADD45a	Abnova	1:4000
MDM2	GeneTex	1:1000
SIP	Santa Cruz Biotechnology	1:1000
XPC	Abcam	1:1000
anti-mouse HRP	Santa Cruz Biotechnology	1:2000
anti-rabbit HRP	Santa Cruz Biotechnology	1:2000
anti-mouse poly-HRP	Thermo Fisher Scientific	1:5000
anti-rabbit poly-HRP	Thermo Fisher Scientific	1:5000

### Statistical analysis

Each experiment was at least performed in triplicate unless otherwise stated and all statistical analyses were performed using Prism^®^ V6 (GraphPad Software, La Jolla, CA, USA). For cisplatin cytotoxicity experiments, dose-effect curves were calculated by non-linear regression based on a four-parameter logistic Hill equation. EC_50_ values were assumed to be log-normally distributed. pEC_50_ values were computed from the turning point of the dose-effect curve for each experiment and the mean of pEC_50_ of all independent experiments was calculated. For the cisplatin accumulation and DNA-adduct formation experiments, means of each independent experiment were calculated and compared between groups by a one-way ANOVA.

To analyze whether gene/protein expression was induced by cisplatin, the significance of a difference between treated and untreated cells was assessed using a one-sample t-test or a one-way ANOVA with Bonferroni's multiple comparisons test as appropriate. When a significant difference was observed, the extent of induction was compared between the cell lines and treatment conditions using a one-way ANOVA with Bonferroni's multiple comparisons test. Differences were considered to be statistically significant at p-values < 0.05.

Author’s notes: mRNA and protein data in the results section are presented relative to untreated control. The individual data points showing the values of untreated controls can be found in Supporting Information (S1–S7 Figs and S8 Fig Data Tables).

## Results

### Cisplatin cytotoxicity

**Fig 1:**
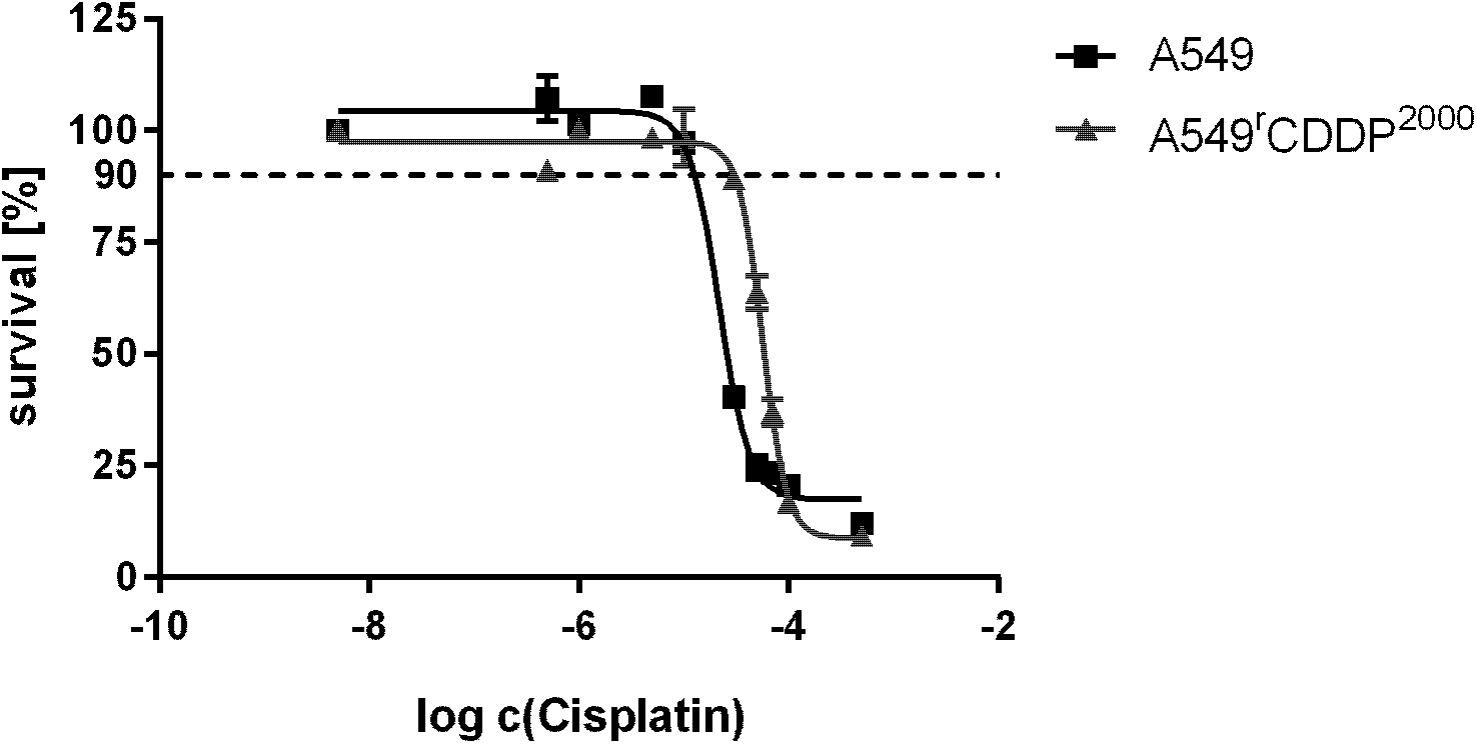
Representative sigmoidal concentration-response curve of cisplatin in A549 and A549^r^CDDP^2000^ cells. Survival is expressed in terms of % compared to untreated cells as mean ± SD.

Cisplatin cytotoxicity was markedly reduced in the A549^r^CDDP^2000^ cells (pEC_50_ = -4.262 ± 0.171; mean ± SD, n = 12) compared to the A549 cell line (pEC_50_ = -4.522 ± 0.144; n = 11) after 24 h treatment. Based on the sigmoidal concentration-response curves, 90% viability concentrations (EC_10_ values) were determined as 11 μM in A549 and 34 μM in A549^r^CDDP^2000^ cells (approx. factor 3) (Fig 1).

### Cellular platinum accumulation

**Fig 2:**
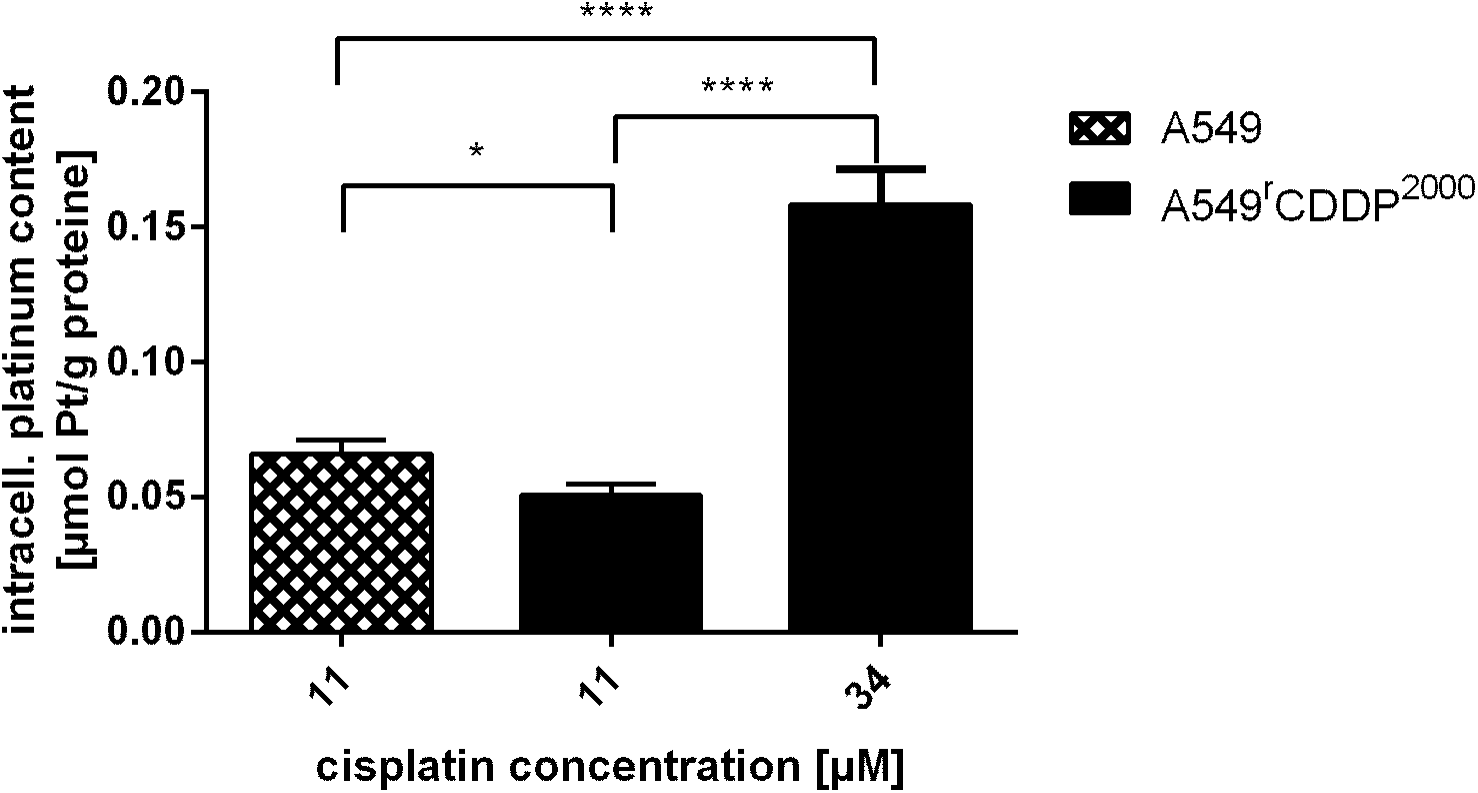
Cellular platinum accumulation. Accumulation of cellular platinum (n ≥ 11) in A549 and A549^r^CDDP^2000^ cells, treated with 11 μM or 34 μM cisplatin and presented as mean ± SEM.

To assess cisplatin accumulation, the intracellular platinum concentration was measured in both cell lines (Fig 2). The intracellular platinum uptake was significantly (p < 0.01) reduced in A549^r^CDDP^2000^ cells (0.051 μmol platinum/g protein, SEM = 0.004; n = 31) compared to A549 cells (0.066 μmol platinum/g protein, SEM = 0.005; n = 33) after treatment of both cell lines with equimolar concentrations of 11 μM cisplatin. After treating the resistant cells with an equitoxic concentration of 34 μM, the accumulated platinum content raised to 0.158 μmol platinum/g protein (SEM = 0.013; n = 29), which was significantly higher (p < 0.0001) than in sensitive cells treated with the equitoxic concentration.

### Cisplatin-DNA adduct formation

**Fig 3:**
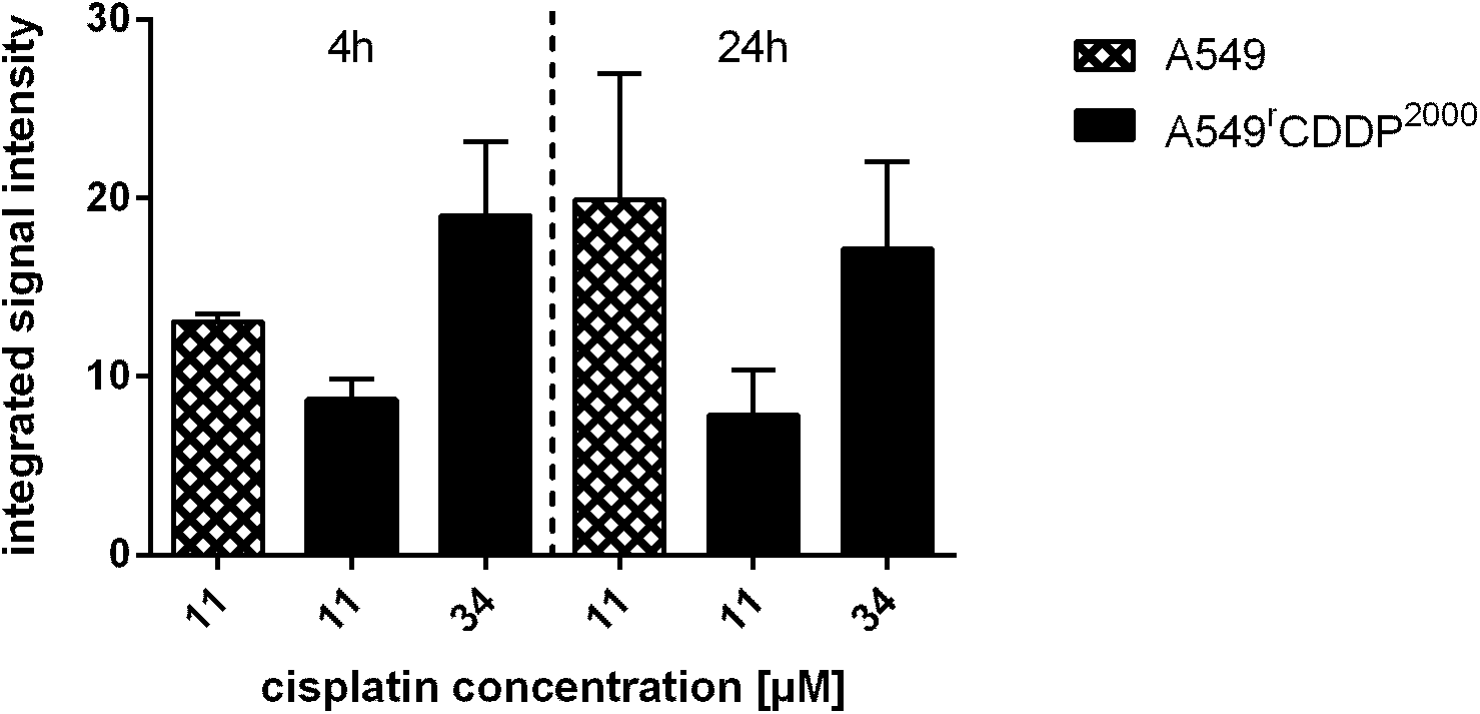
Cisplatin-DNA adduct formation. Content of DNA adducts (n = 3) in A549 and A549^r^CDDP^2000^ cells, treated with 11 μM or 34 μM cisplatin for 4 h and 24 h. Results are related to A549 cells treated 4 h with 11 μM cisplatin and presented as mean ± SEM.

As mentioned above, cisplatin exerts its cytotoxicity through induction of crosslinks on DNA, which is generally seen as the primary DNA damage by the drug. After treatment with equimolar concentration of cisplatin, the A549^r^CDDP^2000^ cells showed a lower level of Pt-DNA adducts. The equitoxic treatment led to an increased adduct formation in A549^r^CDDP^2000^ cells after 4 h treatment compared to A549 cells. After 24 h treatment, resistant cells showed similar DNA-platination as after 4 h, whereas an increase in platinum-DNA adduct formation was observed in sensitive cells over time. These data indicate that A549^r^CDDP^2000^ cells acquired resistance mechanisms that reduce DNA platination, e.g. by repair mechanisms, in comparison to A549 cells. At equitoxic concentrations, cellular platinum accumulation was about 3-fold higher in A549^r^CDDP^2000^ cells than in A549 cells. This higher intracellular platinum exposure did not result in enhanced cisplatin-DNA adduct formation (Fig 3).

### Cell cycle analysis

**Fig 4:**
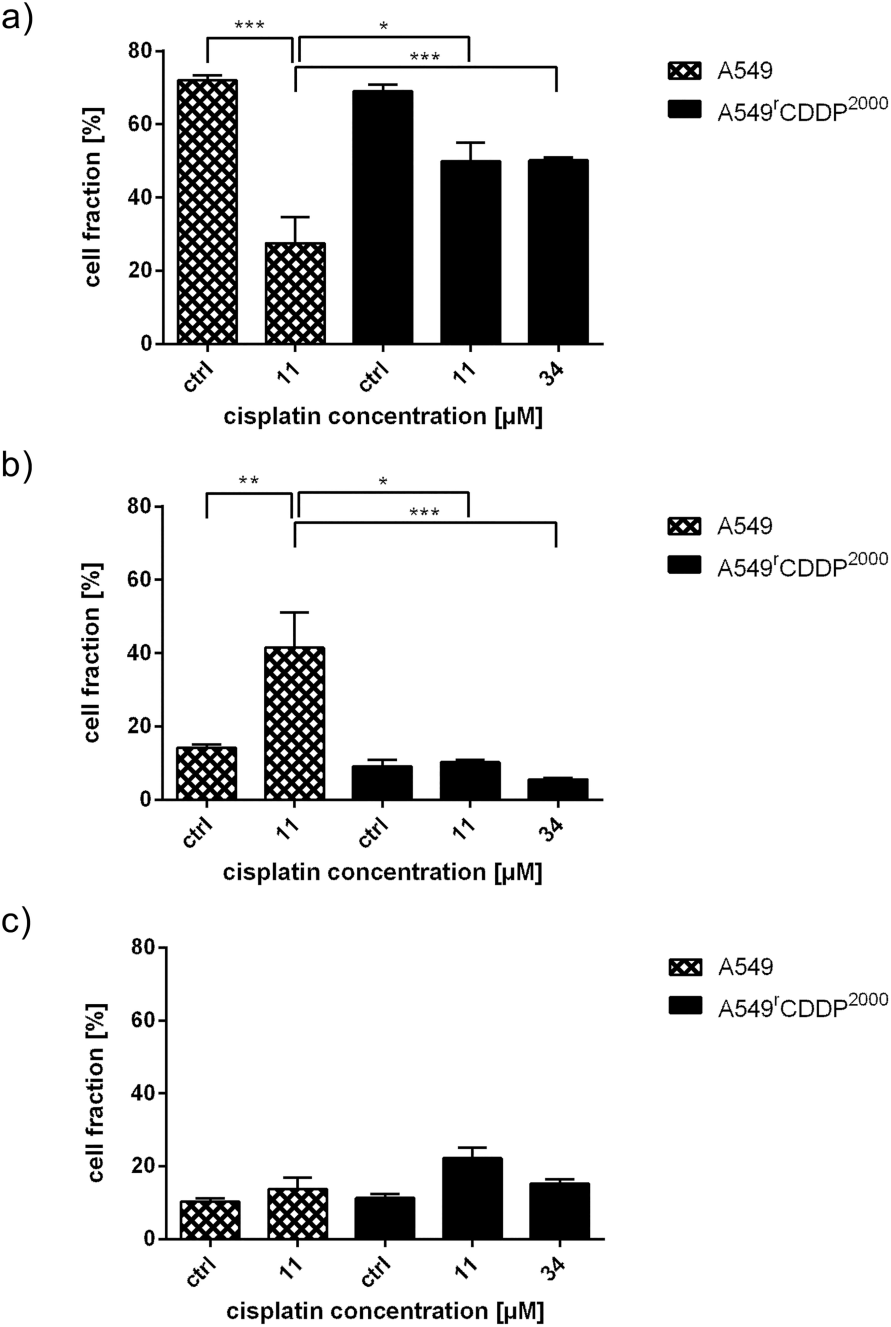
Cell cycle distribution in A549 and A549^r^CDDP^2000^ cells. Cell fraction of the flow cytometric cell cycle analysis is presented in % of total cell population in a) G_1_/G_0_-phase, b) G_2_/M-phase and c) S-phase in A549 and A549^r^CDDP^2000^ cells as untreated controls or after treatment with 11 μM or 34 μM cisplatin presented as mean ± SEM.

A549 cells treated with 11 μM cisplatin showed a significant decrease in G_1_/G_0_ phase compared to that of equitoxic (34 μM) and equimolar (11 μM) cisplatin treatment in A549^r^CDDP^2000^ cells (Fig 4A). A more striking difference was observed in the G_2_/M-phase, where A549 cells treated with 11 μM cisplatin showed a significant level of cell cycle arrest compared to that of A549^r^CDDP^2000^ cells, treated with either equimolar or equitoxic concentrations (Fig 4B). Thus, A549^r^CDDP^2000^ cells seem to have a mechanism to suppress DNA damage-induced G_2_/M-arrest. The fraction of cells in the S-phase did not differ among cell lines and treatments (Fig 4C).

### Apoptosis induction

**Fig 5:**
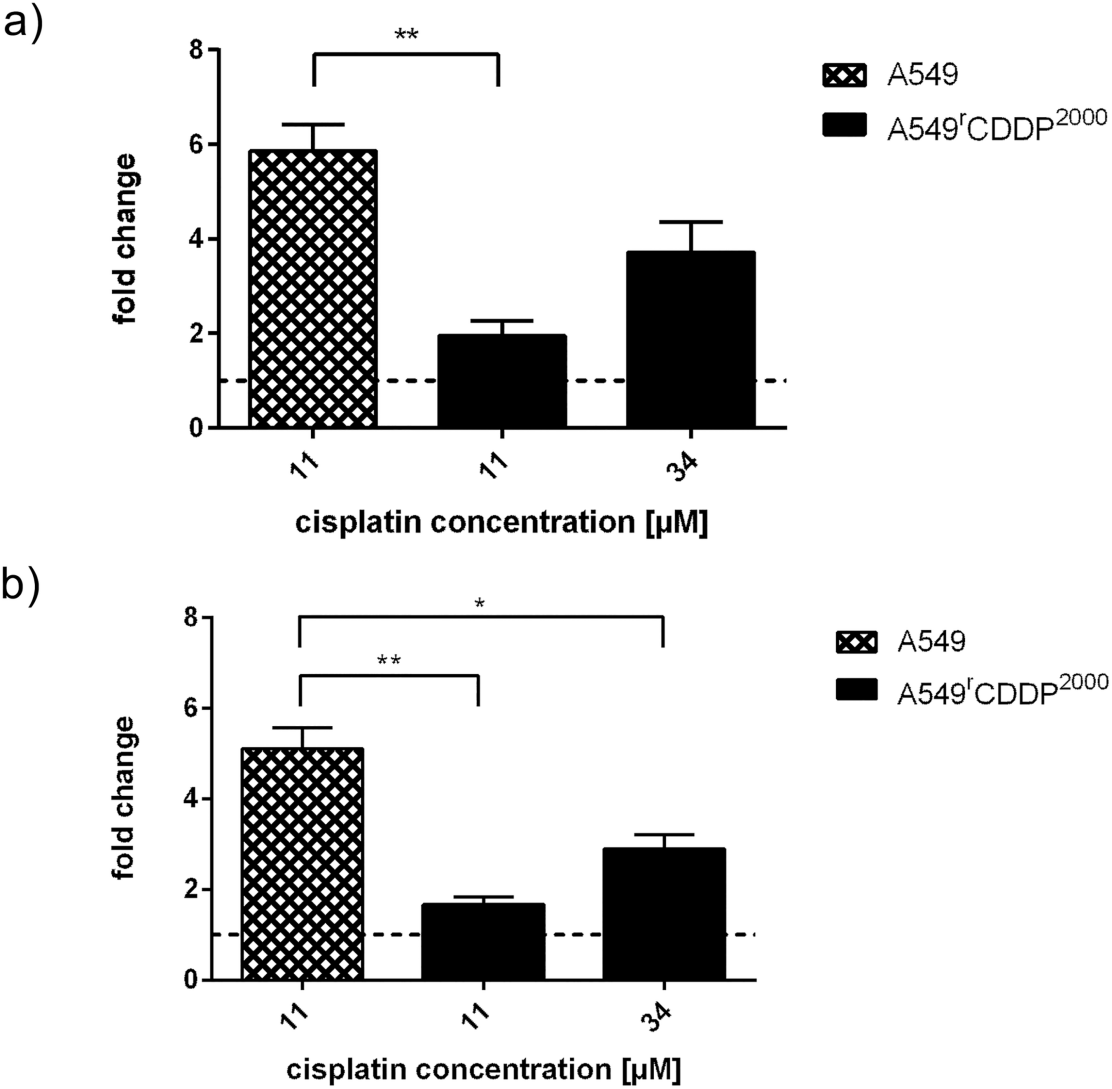
Apoptosis induction in A549 cells. **A549**^**r**^**CDDP**^**2000**^ **cells.** Apoptosis analysis with a) FITC Annexin (n ≥ 3) and b) cell count in SubG_1_-phase (n = 3) presented as fold change related to respective untreated controls in A549 and A549^r^CDDP^2000^ cells as mean ± SEM.

After treatment with 11 μM cisplatin, apoptosis was markedly induced in A549 cells. A549^r^CDDP^2000^ cells exhibited a statistically significant reduction in the number of apoptotic cells in response to treatment with equimolar concentrations of cisplatin compared to A549 cells. A549^r^CDDP^2000^ cell treatment with 34 μM resulted in a tendency to increase the number of apoptotic cells compared to 11 μM cisplatin exposure, but remained lower than the number of apoptotic cells in sensitive cells treated with the equitoxic concentration of 11 μM (Fig 5A). Similar results were obtained by the quantification of the number of cells in SubG_1_-phase (Fig 5B).

### Response of the p53 system

**Fig 6:**
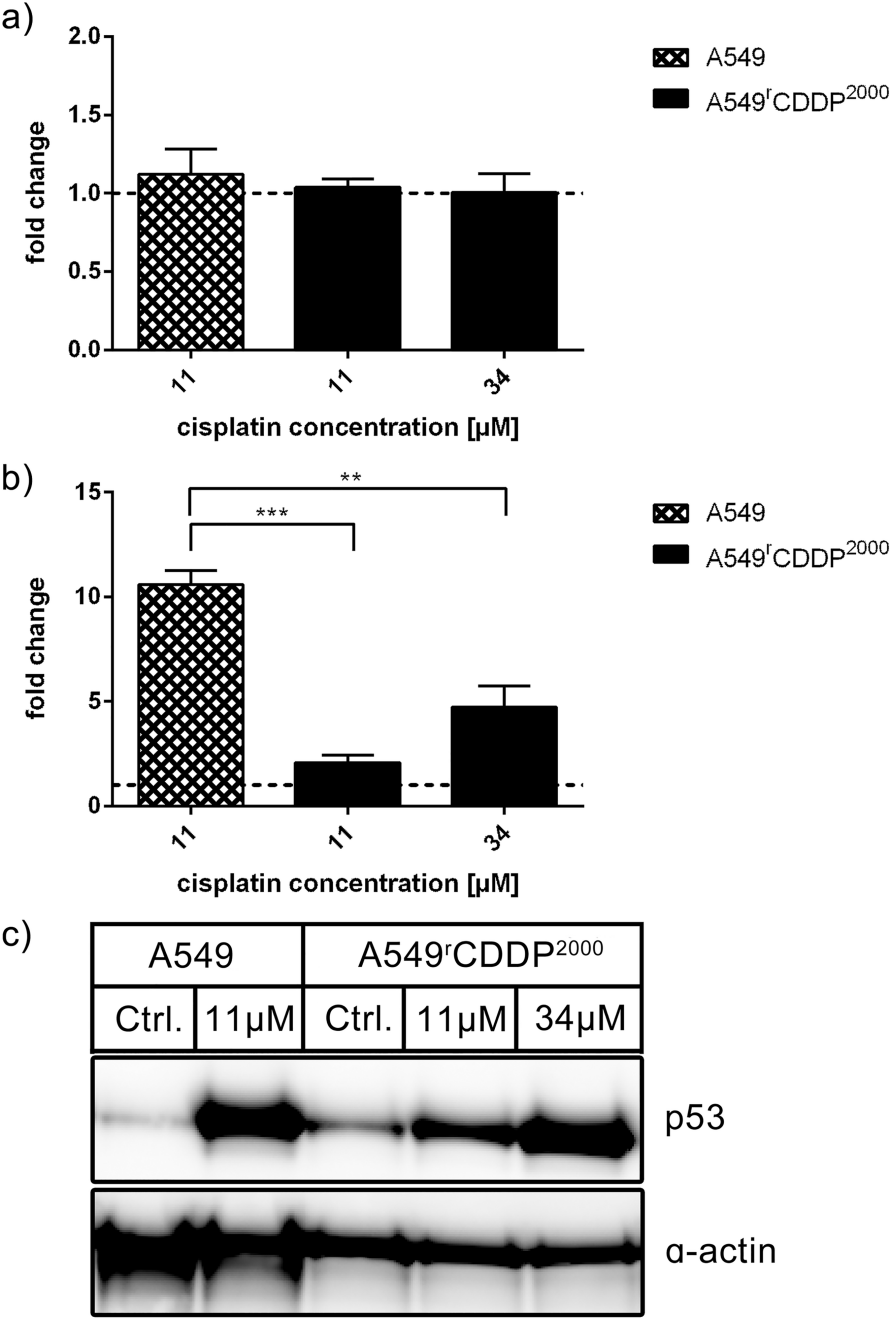
p53 mRNA and protein analysis. Analysis of p53 in a) RT-PCR (n = 3) and b) Western Blot (n = 3) as fold change relative to untreated control in A549 and A549^r^CDDP^2000^ cells, presented as mean ± SEM with c) one representative western blot showing A549 untreated, A549 treated with 11 μM cisplatin, A549^r^CDDP^2000^ untreated, A549^r^CDDP^2000^ treated with 11 μM cisplatin and A549^r^CDDP^2000^ treated with 34 μM cisplatin.

p53 plays a major role in DNA damage response, apoptosis and cell cycle regulation. Which path the cell enters is mainly dependent on which target genes of p53 are affected. p53 can be activated in a number of different ways like phosphorylation and acetylation with specific complex patterns of modifications assumed to trigger downstream effects [13]. Here we focused on investigating important key players of the downstream signaling of p53 at the mRNA and protein level in order to determine differences in genes and proteins affected by p53 signaling in response to cisplatin treatment between A549 and A549^r^CDDP^2000^ cells. As expected because of a minor role of changes in p53 transcription and mainly regulation on protein level [14], p53 was not regulated at the mRNA level (Fig 6A) but showed a significantly (p < 0.01) higher baseline level in resistant cells compared to sensitive ones. There was a significant accumulation of the protein in fold change in cisplatin-treated sensitive cells compared to resistant ones treated with 11 μM cisplatin (p < 0.01) or with 34 μM cisplatin (p < 0.001) (Fig 6B and 6C). Absolut data revealed that only treatment in sensitive cells resulted in a significant increase in protein accumulation. Equitoxic cisplatin treatment induced a similar increase of p53 protein expression in A549 and A549^r^CDDP^2000^ cells (S1 Fig).

**Fig 7:**
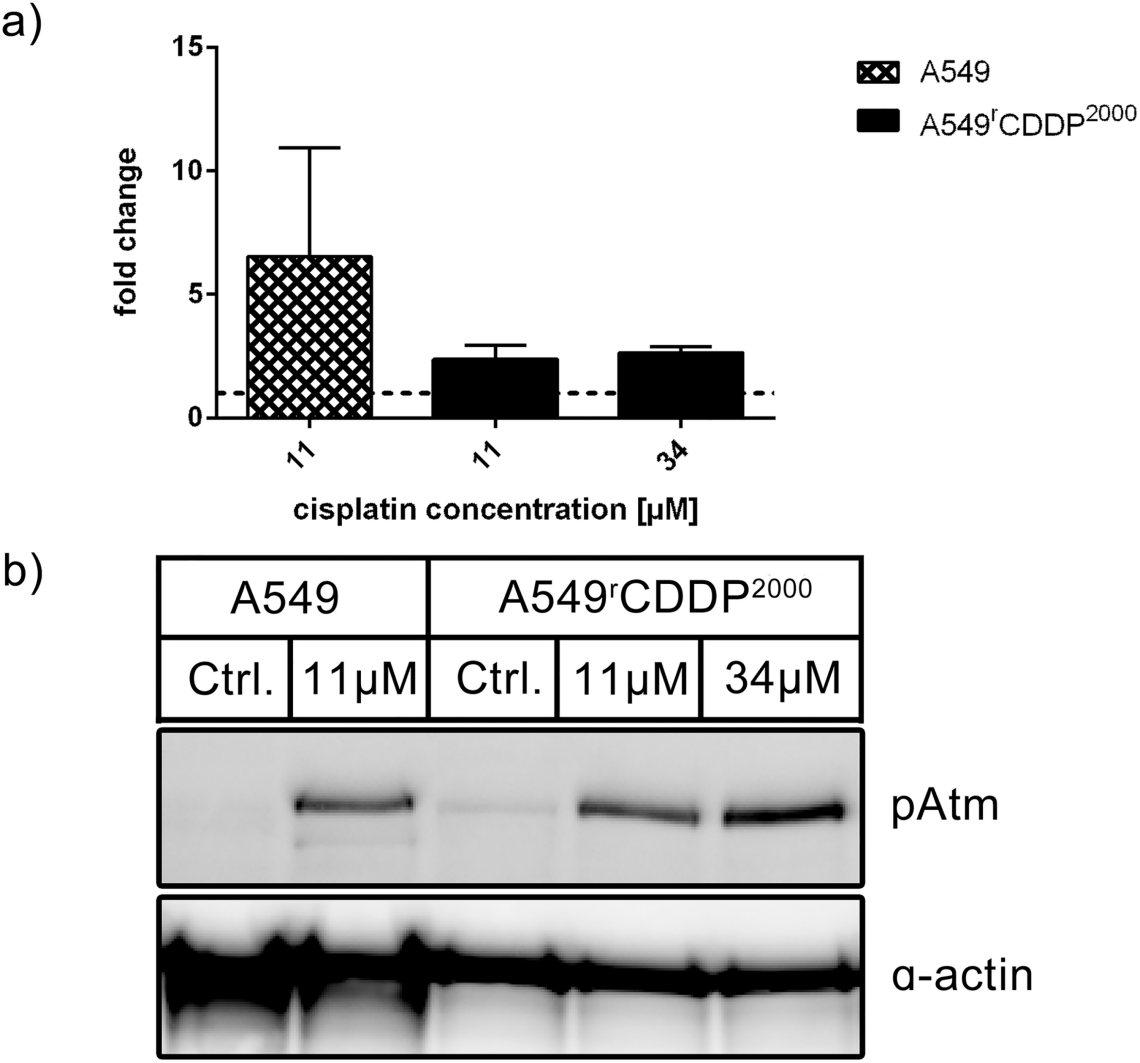
pATM protein analysis. Western Blot Analysis of pAtm (n = 3) a) as fold change relative to untreated control normalized to the housekeeper α-actin in A549 and A549^r^CDDP^2000^ cells, presented as mean ± SEM with b) one representative western blot showing A549 untreated, A549 treated with 11 μM cisplatin, A549^r^CDDP^2000^ untreated, A549^r^CDDP^2000^ treated with 11 μM cisplatin and A549^r^CDDP^2000^ treated with 34 μM cisplatin.

Upstream of p53, the activated DNA damage recognition protein Ataxia Telangiectasia mutated (Atm) protein showed a higher increase in relative protein level in sensitive cells compared to A549^r^CDDP^2000^ cells (Fig 7A and 7B). This difference in increase of pATM was not significant. However, in sensitive cells the increase in pATM expression was significant compared to control (p < 0.05) after treatment with 11 μM cisplatin whereas it was not significant in resistant cells (S2 Fig).

**Fig 8:**
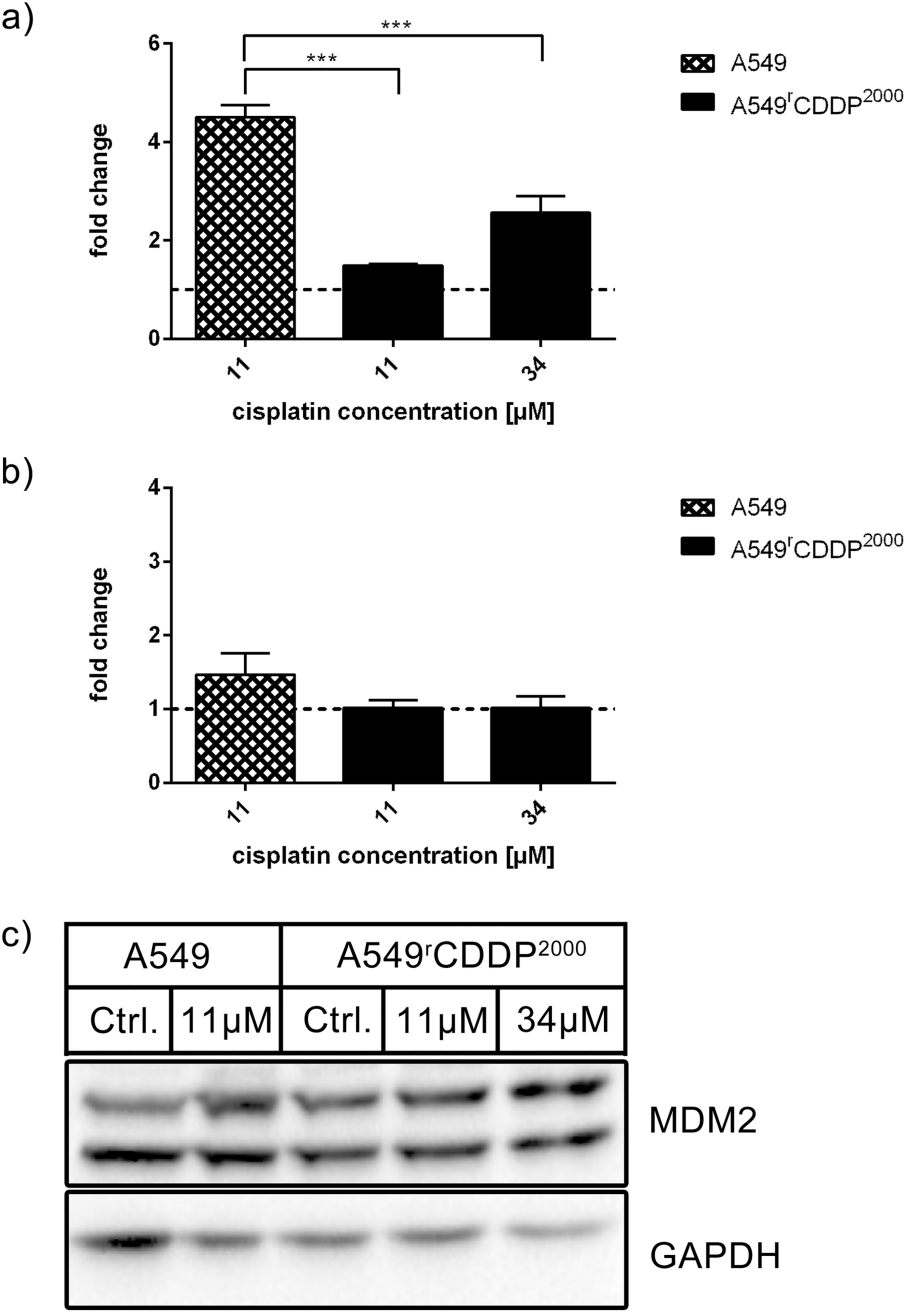
MDM2 mRNA and protein analysis. Analysis of MDM2 in a) RT-PCR (n = 3) and b) Western Blot (n = 3) as fold change relative to untreated control in A549 and A549^r^CDDP^2000^ cells, presented as mean ± SEM with c) one representative western blot showing A549 untreated, A549 treated with 11 μM cisplatin, A549^r^CDDP^2000^ untreated, A549^r^CDDP^2000^ treated with 11 μM cisplatin and A549^r^CDDP^2000^ treated with 34 μM cisplatin.

Mouse double minute 2 homolog (MDM2) protein, a p53 target and endogenous p53 antagonist was significantly up-regulated on mRNA level (p < 0.05) after 24 h treatment with equitoxic cisplatin concentrations in both cell lines (S3 Fig). mRNA levels were comparable in sensitive and resistant cells. The level of induction in fold change compared to control however showed significantly higher levels in sensitive cells compared to equimolar (p < 0.001) and equitoxic (p < 0.001) treatment in resistant cells (Fig 8A). No significant changes in MDM2 protein expression were observed in both cell lines after cisplatin treatment (Fig 8B and 8C).

**Fig 9:**
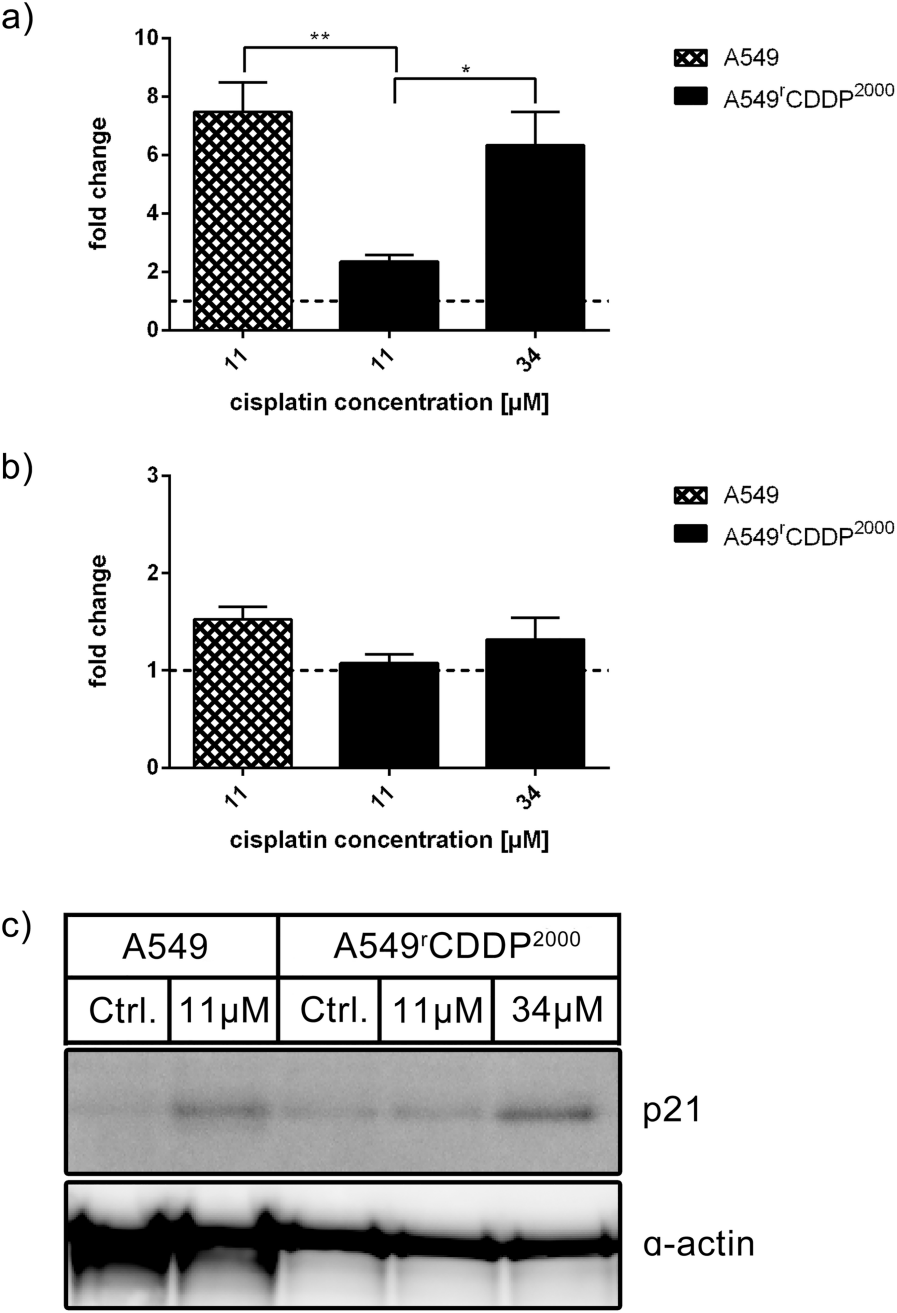
p21 mRNA and protein analysis. Analysis of p21 in a) RT-PCR (n = 3) and b) Western Blot (n = 3) as fold change relative to untreated control in A549 and A549^r^CDDP^2000^ cells, presented as mean ± SEM with c) one representative western blot showing A549 untreated, A549 treated with 11 μM cisplatin, A549^r^CDDP^2000^ untreated, A549^r^CDDP^2000^ treated with 11 μM cisplatin and A549^r^CDDP^2000^ treated with 34 μM cisplatin.

Downstream of p53, p21 plays a central role in cell cycle control, regulating both, G_1_/S and G_2_/M cell cycle checkpoints. Therefore, this protein needs to be investigated to understand differences between cisplatin-sensitive and resistant cells with regards to cell cycle arrest. The mRNA of p21, a protein involved in regulation of the cell cycle, was significantly higher in fold change after 11 μM treatment in sensitive cells (p < 0.01) and after 34 μM treatment in resistant cells (p < 0.05) compared to 11 μM treatment in resistant cells (Fig 9A). It was significantly (p < 0.05) up-regulated on mRNA level upon all treatment conditions and in both cell lines (S4 Fig). On protein level no significant changes were observed (Fig 9B and 9C).

**Fig 10:**
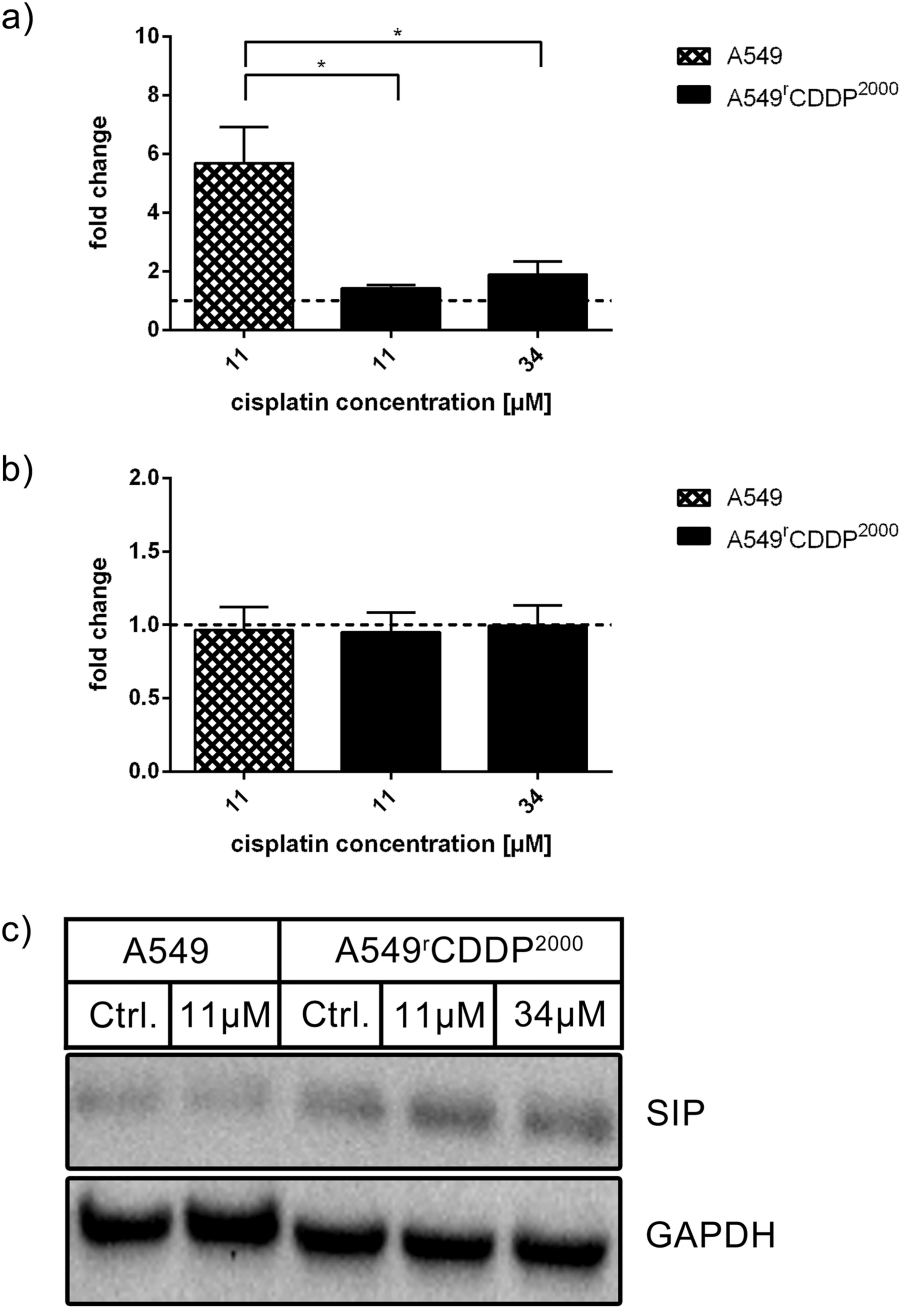
SIP mRNA and protein analysis. Analysis of SIP in a) RT-PCR (n = 3) and b) Western Blot (n = 3) as fold change relative to untreated control in A549 and A549^r^CDDP^2000^ cells, presented as mean ± SEM with c) one representative western blot showing A549 untreated, A549 treated with 11 μM cisplatin, A549^r^CDDP^2000^ untreated, A549^r^CDDP^2000^ treated with 11 μM cisplatin and A549^r^CDDP^2000^ treated with 34 μM cisplatin.

Relative mRNA levels of SIP, a stress-induced protein and another upstream activator (cofactor) of p53, were significantly (p < 0.05) increased in sensitive cells after treatment with cisplatin compared to resistant cells (Fig 10A). In A549^r^CDDP^2000^ cells, SIP expression was not induced by cisplatin treatment in contrast to a significant increase in sensitive cells (S5 Fig). These results were not transferred to the protein level, where no regulation of protein expression was seen (Fig 10B and 10C). However, basal levels of SIP were significantly higher in the resistant cell line (S5 Fig).

**Fig 11:**
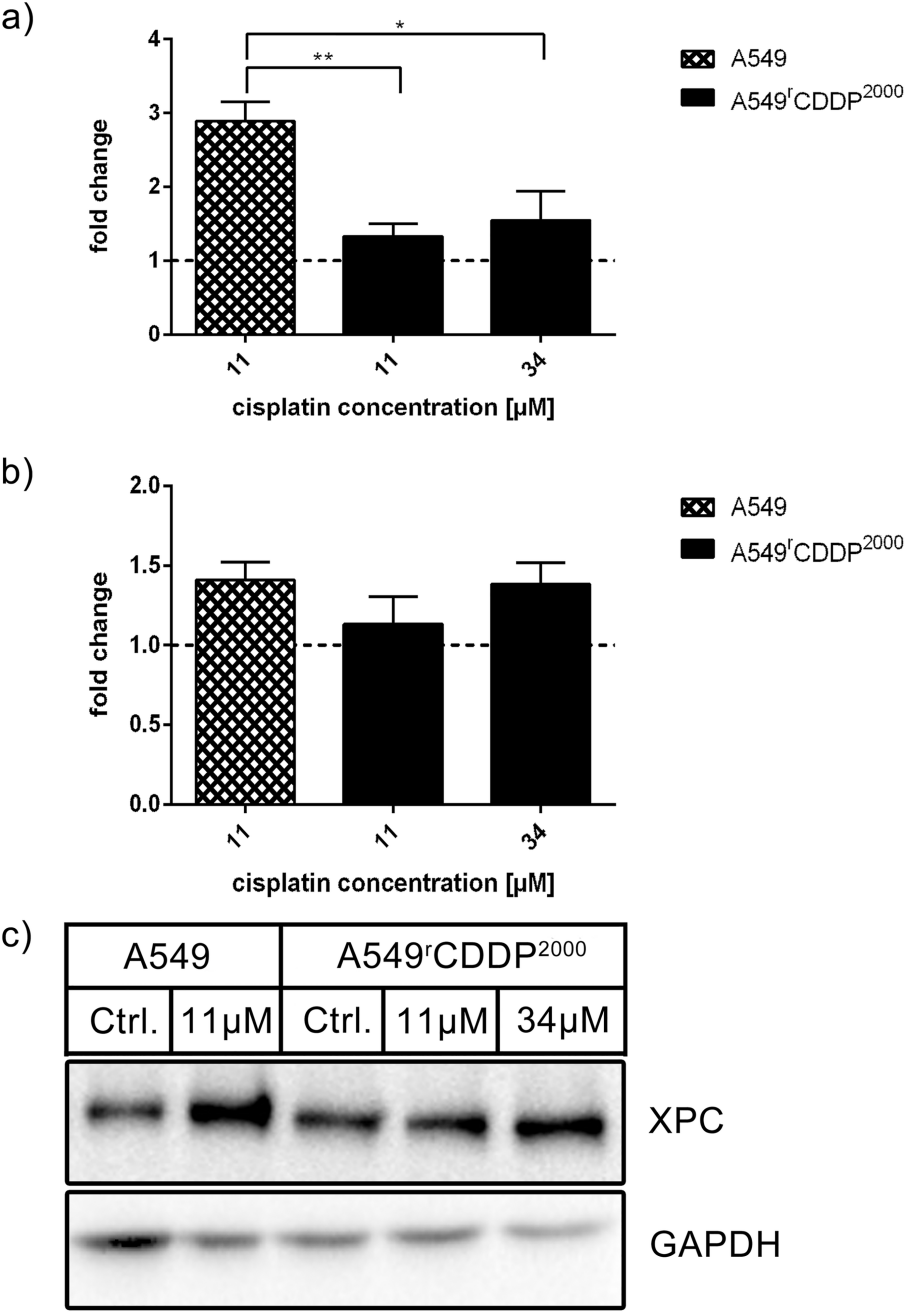
XPC mRNA and protein analysis. Analysis of XPC in a) RT-PCR (n = 3) and b) Western Blot (n = 3) as fold change relative to untreated control in A549 and A549^r^CDDP^2000^ cells, presented as mean ± SEM with c) one representative western blot showing A549 untreated, A549 treated with 11 μM cisplatin, A549^r^CDDP^2000^ untreated, A549^r^CDDP^2000^ treated with 11 μM cisplatin and A549^r^CDDP^2000^ treated with 34 μM cisplatin.

Xeroderma pigmentosum, complementation group C (XPC) mRNA, which encodes a member of the nucleotide excision repair system and is a downstream effector of p53, was significantly up-regulated in fold change of control in sensitive cells after cisplatin treatment (Fig 11A). The induction of XPC mRNA expression was significantly stronger in sensitive cells than in resistant cells, when treated with equimolar (p < 0.01) and equitoxic (p < 0.05) concentrations (S6 Fig). On protein level, XPC showed no significant changes after treatment with cisplatin (Fig 11B and 11C).

**Fig 12:**
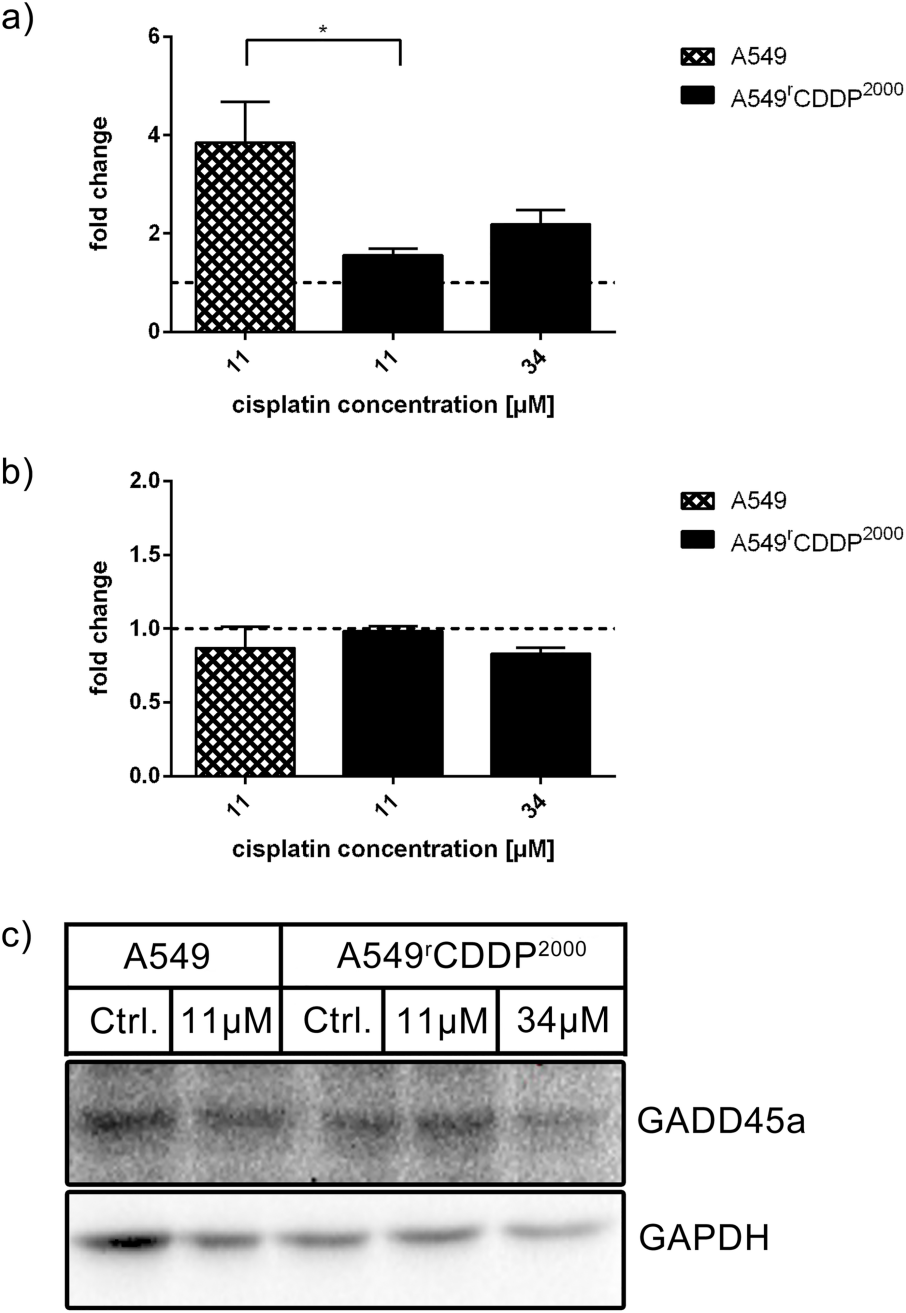
GADD45a mRNA and protein analysis. Analysis of GADD45a in a) RT-PCR (n = 3) and b) Western Blot (n = 3) as fold change relative to untreated control in A549 and A549^r^CDDP^2000^ cells, presented as mean ± SEM with c) one representative western blot showing A549 untreated, A549 treated with 11 μM cisplatin, A549^r^CDDP^2000^ untreated, A549^r^CDDP^2000^ treated with 11 μM cisplatin and A549^r^CDDP^2000^ treated with 34 μM cisplatin.

Growth arrest and DNA-damage-inducible protein GADD45 alpha (GADD45a), which in general is a downstream effector of p53 with impact on checkpoint kinases and an inducer of cell cycle arrest, showed significantly different expression upon treatment with 11 μM cisplatin in A549 cells compared to A549^r^CDDP^2000^ cells treated with equimolar concentration (Fig 12A). On the protein level, however no significant differences between treated and untreated cells were observed (Fig 12B and 12C).

### Proposed model of resistance-associated signaling alterations

**Fig 13:**
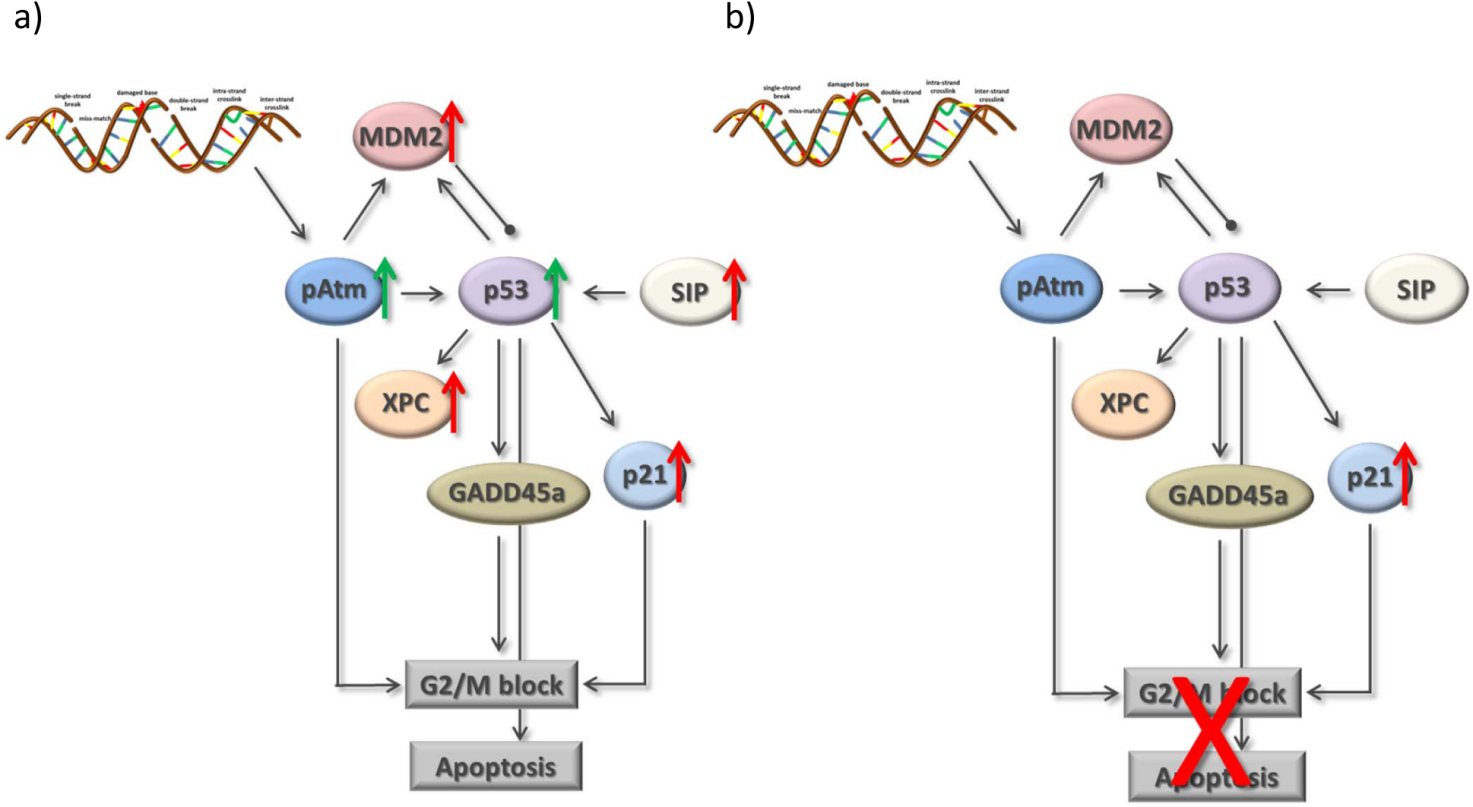
Signalling model for reduction of G_2_/M arrest in A549^r^CDDP^2000^ cells. Preliminary signaling model to explain the lack of a G_2_/M arrest in resistant cells indicating significant protein changes after treatment comparing a) A549 and b) A549^r^CDDP^2000^ cells based on mRNA data (red arrows) or protein data (green arrows).

Based on the results described above, a preliminary model reflecting the changes in signaling after treatment with cisplatin in A549 and in A549^r^CDDP^2000^ cells could be constructed (Fig 13). The model includes relevant genes, which are activated by cisplatin-induced DNA damage. The changes associated to cisplatin treatment were compiled to explain the observed differences in cell cycle analysis, DNA-platination and cellular cisplatin accumulation. Based on the data from mRNA analysis, the resulting changes are indicated with red arrows. The green arrows indicate protein up-regulation. These specific alterations lead to inhibition of cell cycle arrest and apoptosis in A549 cells after cisplatin treatment in contrast to A549^r^CDDP^2000^ cells.

## Discussion

We intended to elucidate molecular key events shading more light on the potential mechanistic background of the cellular development of cisplatin resistance. Cisplatin treatment leads to cisplatin-DNA adducts and DNA crosslinks. Major cellular responders to Pt-DNA adduct formation are pATM and p53. We therefore focused our investigation on genes and proteins which are potential effectors of these proteins and also assumed to be involved in cell cycle and apoptosis control. MDM2 was considered of importance due to its major activity in p53 homoeostasis and SIP for its kinase activity on p53. p21 is an effector of p53 and a major cell cycle checkpoint control protein [15]. XPC was in our interest as it is involved in DNA repair but mainly also because it has been suggested to predict treatment outcome in patients with NSCLC [16,17].

### Cell system

To elucidate the molecular mechanisms underlying acquired cisplatin resistance, we investigated the NSCLC cell line A549 and its cisplatin-resistant sub-line A549^r^CDDP^2000^. The resistant cells displayed a twofold higher EC_50_ value, lower apoptosis level, as well as alterations in intracellular platinum accumulation and DNA platination. The results displayed here are consistent with the two-fold lower sensitivity of resistant tumor cells to cisplatin in clinical studies for ovarian cancer [18] and the resistance in A549 cells measured by Yang et al. [19].

The EC_10_ concentrations used in the experiments were precautionary to prevent effects superimposing resistance mechanisms. They were comparable to the clinically attainable concentrations [20,21]. To compensate the effects of altered influx or efflux of cisplatin in resistant cells, equitoxic concentrations were studied showing similar Pt-DNA adduct formation but differences in drug accumulation. We previously demonstrated in other cell lines [22] that reduced cisplatin accumulation may be one source of chemoresistance, which needs to be interpreted in relation to DNA platination. At equimolar concentrations, platinum-DNA adduct formation was not significantly lower in A549^r^CDDP^2000^ cells after 4 h treatment compared to sensitive cells and increased over time in A549 cells only. Equitoxic concentrations led to a subproportional increase of DNA-adduct levels in resistant cells compared to the intracellular platinum accumulation. After 24 h treatment with equitoxic concentrations, DNA adduct levels were similar in A549 and A549^r^CDDP^2000^ cells suggesting that resistant cells exhibit a higher DNA-repair capacity than sensitive cells, as intracellular platinum accumulation was significantly higher in resistant cells. Other explanations for similar DNA platination levels despite higher cellular platinum accumulation in A549^r^CDDP^2000^ at equitoxic concentrations are increased sequestration in vesicles and an increased drug inactivation compared to the sensitive cells. Glutathione or for instance glutathione-like enzymes are known to act as detoxification agents for cisplatin [23–28].

### DNA damage and repair

Although equitoxic cisplatin concentrations resulted in similar amount of Pt-DNA adducts in sensitive and resistant cells, the cellular response showed significant differences. Apoptosis was only induced in sensitive cells pointing to an altered DNA-damage response in resistant cells. It was previously shown that resistant NSCLC cells have a higher repair capacity [29,30]. The impact of this phenomenon leading to resistance was documented in several studies [26,31,32]. This is in agreement with our results concerning phosphorylation of ATM, which is responsible for the recognition of DNA double-strand breaks, finally leading to a G_2_/M arrest in sensitive cells. As expected from literature, this protein shows nearly no activity in untreated control cells. Bakkenist et al. investigated the activation of ATM and reported that basal, dimerized ATM is inactive. While total ATM was constant, pATM level rose after DNA damage by radiation. Only pATM after monomerization was capable of activating other proteins [33].

These cellular events further lead to activation of SIP which is a cofactor of p53. SIP is capable of modulating p53 activity and leads to the expression of antiproliferative and proapoptotic target genes of p53, like p21 [34,35]. This signaling pathway is activated after several different stress inducers in tumor cells [34,36,37]. Activation of SIP by cisplatin promoting cell death was also shown in other cell lines [38]. In line with this work SIP is activated after treatment with cisplatin on mRNA level, but only in sensitive cells.

Furthermore, DNA damage tolerance may contribute to cisplatin resistance of A549^r^CDDP^2000^ cells. Cisplatin treatment significantly increased mRNA level of XPC, a protein downstream of p53 and crucial for DNA damage recognition, in sensitive cells. This suggests higher activation of the global genome repair pathway and therefore a lower tolerance to cisplatin-DNA adducts in A549. Beside its role in DNA damage recognition, XPC plays a major role in altering cell cycle after treatment with cisplatin. In XPC-deficient cell lines, the p53 pathway is altered and cell cycle arrest, DNA repair and apoptosis are attenuated [39]. XPC-deficient transgenic mice are highly predisposed to several types of cancer [40] and XPC/GADD45a knockout in mice leads to development of lung tumors [41]. XPC expression is also reduced in the tumor tissue of resistant patients compared to normal lung tissue [42]. Additionally, reduced XPC mRNA is suggested to predict a poor outcome for patients with NSCLC [16]. Weaver et al. showed that XPC correlates with chemoresistance in NSCLC [17]. GADD45a, also enhancing NER [43], was induced in sensitive cells after treatment in contrast to the resistant cells were no significant up-regulation was found. This may support the hypothesis that NER response is reduced in resistant cells. Overall, it can be seen that sensitive cells show stronger reactions in mRNA expression than resistant cells. They again seem to be more robust.

### Cell cycle alterations

Ataxia telangiectasia fibroblasts lack G_1_ and G_2_ phase checkpoint functions indicating that activation of ATM to pATM is crucial for cell cycle control [44]. This is in agreement with reduced pATM and G_2_/M arrest in resistant vs. sensitive cells.

GADD45a is involved in cell cycle regulation and responsible for a G_2_/M arrest to enhance DNA repair [45]. GADD45a mRNA expression was increased in HDFa cells after genistein treatment corresponding to cell cycle arrest [46]. GADD45a may contribute to the G_2_/M-phase cell cycle arrest in A549 cells in response to treatment with cisplatin. Fold change of GADD45a mRNA was significantly different in A549^r^CDDP^2000^ cells treated with 11 μM cisplatin compared to sensitive cells after treatment. This, however, did not translate into a significant difference in GADD45a protein levels at the time point studied. Reduced expression of GADD45a has been associated with poor survival in esophageal cancer patients [47]. The cell cycle alterations are in agreement with previously published work [48], showing that tumor initiating cells are prone to less G_2_/M-arrest after DNA-damaging treatment. Horibe et al. showed that cisplatin resistance is linked to loss of G_2_/M arrest in cisplatin-resistant cells [49]. The p53 target gene product p21 that induces cell cycle arrest in G_2_/M-phase was up-regulated in sensitive and resistant cells after treatment with cisplatin. Comparing the fold change to control, sensitive and resistant cells treated with equitoxic concentrations resulted in a significantly higher expression of p21 than in resistant cells treated with 11 μM cisplatin. This suggests a p53-mediated cell cycle arrest in sensitive cells that is inactive at the 11 μM in cisplatin-resistant cells. Activation of this mechanism needs much higher doses in resistant cells. We assume that this adaptation to cisplatin treatment is part of the mechanism of resistance in A549^r^CDDP^2000^ cells.

MDM2 ubiquitinates p53 and regulates its activity and degradation in an autoregulatory feedback loop. MDM2 was significantly activated at the mRNA level in cisplatin-treated cells at equitoxic concentrations. The extent of activation in A549 cells was significantly higher compared to A549^r^CDDP^2000^ cells. On protein level, no significant differences could be observed. One result of ubiquitination by MDM2 is destabilization of p53, diminishing the reservoir of p53 which could be easily and quickly activated if needed. This consecutive existing balance between MDM2 and ubiquitinated p53 would be disturbed if MDM2 protein levels are altered rapidly with high amplitudes, e.g. massive expression in short time. In consequence, a significant reduction of MDM2 would lead to an over-imposing activity of p53, which is physiologically unfavorable in resistant tumor cells. To avoid this, only moderate changes take place in this equilibrium. As MDM2 is also a downstream transcriptional target of p53 [50], p53 activation may be altered in resistant cells. In the future, we shall have a closer look at the activation status of p53 after treatment with cisplatin, as this may be one key difference in p53 regulation.

### Starting point for a systems approach

Based on the results presented above we have developed a signaling model (Fig 13), which displays possible connections between the key players of cellular response to cisplatin exposure. This model reveals some mechanisms accounting for a different reaction of the sensitive and resistant NSCLC cells to the treatment. It provides an overview of the possible roles of several cellular proteins; however, it represents only a small part of the whole picture inside the cell. Results from mRNA level could not necessarily be transferred to the protein level. This could be a matter of the time point of measurement. pAtm and p53 are already activated on protein level in sensitive cells, introducing the G_2_/M arrest. mRNA activation takes place at an earlier stage. p53 is now already capable of acting as a transcription factor to activate the other proteins in the signaling model. These are consequently activated on mRNA level but possibly not yet on protein level. A weak correlation between mRNA and protein expression has been reported earlier. Possible mechanisms accounting for the lack of correlation include: (1) post-transcriptional factors, (2) post-translational parameters, as well as (3) noise and experimental error. It appears difficult to assess whether translation efficiency or protein half-life most prominently impacts the correlation between mRNA and protein abundance [51]. An analysis in a space- and time-dependent manner should be applied in the future and may help to resolve this discrepancy.

We assume that there may not be one key driver for resistance development. Loss of activation of pATM and p53 seems to be a major step to tolerance against cisplatin in these cells which, however, seems not surprising given the DNA-adduct formation of cisplatin. There may be more than one or two major key regulators for activation of these two proteins. Moreover, transcription of the downstream effectors as shown for p21 is not exclusively regulated by p53. It seems to be a complex network of different protein interactions that lead to or omit a cellular response to cisplatin treatment. The model presented here is thus not comprehensive and can be extended by further players. Nevertheless, it serves as a good starting point for a systems pharmacology approach aiming at getting a full picture of protein interactions in the intracellular signaling network.

### Conclusion

A549 and A549^r^CDDP^2000^ cells differ in their signaling response to cisplatin treatment. p53 and pATM are significantly induced only in sensitive cells on protein level. MDM2, XPC, SIP, p21, and GADD45a are differently induced on mRNA level in resistant cells compared to sensitive ones, where these key players of the downstream signaling pathways lead to G_2_/M arrest. In the future, knock-out experiments should reveal connections between genes and proteins displayed in the preliminary model. However, as this model most probably only reflects a small part of the network, identification of more key players is needed before employing knock-out experiments. The proposed model of resistance-associated signaling alterations serves as a starting point for a systems pharmacology approach.

## Supporting information

S1 Fig Analysis of p53. Analysis of p53 in a) RT-PCR (n = 3) as individual data points presented as mean ± SEM and b) Western Blot (n = 3) as integrated signal intensity normalized to the housekeeper α-actin in A549 and A549^r^CDDP^2000^ cells, presented as mean ± SEM.
(TIF)

S2 Fig Analysis of pAtm. Western Blot Analysis of pAtm (n = 3) a) as integrated signal intensity normalized to the housekeeper α-actin in A549 and A549^r^CDDP^2000^ cells, presented as mean ± SEM.
(TIF)

S3 Fig Analysis of MDM2. Analysis of MDM2 in a) RT-PCR (n = 3) as individual data points presented as mean ± SEM and b) Western Blot (n = 3) as integrated signal intensity normalized to the housekeeper GAPDH in A549 and A549^r^CDDP^2000^ cells, presented as mean ± SEM.
(TIF)

S4 Fig Analysis of p21. Analysis of p21 in a) RT-PCR (n = 3) as individual data points presented as mean ± SEM and b) Western Blot (n = 3) as integrated signal intensity normalized to the housekeeper α-actin in A549 and A549^r^CDDP^2000^ cells, presented as mean ± SEM.
(TIF)

S5 Fig Analysis of SIP. Analysis of SIP in a) RT-PCR (n = 3) as individual data points presented as mean ± SEM and b) Western Blot (n = 3) as integrated signal intensity normalized to the housekeeper GAPDH in A549 and A549^r^CDDP^2000^ cells, presented as mean ± SEM.
(TIF)

S6 Fig Analysis of XPC. Analysis of XPC in a) RT-PCR (n = 3) as individual data points presented as mean ± SEM and b) Western Blot (n = 3) as integrated signal intensity normalized to the housekeeper GAPDH in A549 and A549^r^CDDP^2000^ cells, presented as mean ± SEM.
(TIF)

S7 Fig Analysis of GADD45a. Analysis of GADD45a in a) RT-PCR (n = 3) as individual data points presented as mean ± SEM and b) Western Blot (n = 3) as integrated signal intensity normalized to the housekeeper GAPDH in A549 and A549^r^CDDP^2000^ cells, presented as mean ± SEM.
(TIF)

S8 Fig Tables with individual data values. Tables with individual data values analysed for preparation of all figures in this manuscript.
(DOCX)

S9 Fig Western Blot of α-Actin for p53 pATM and p21 normalization. Western Blot of α-Actin in A549 and A549rCDDP^2000^ cells. Lanes 1, 6, 11,: A549 untreated; lanes 2, 7, 12, A549 cells treated with 11 μM cisplatin; lanes 3, 8, 13, A549rCDDP^2000^ untreated; lanes 4, 9, 14, A549rCDDP^2000^ treated with 11 μM cisplatin; lanes 5, 10, 15, A549rCDDP^2000^ treated with 34 μM cisplatin.
(TIF)

S10 Fig Western blot p53. Western Blot of p53 in A549 and A549rCDDP^2000^ cells. Lanes 1, 6, 11,: A549 untreated; lanes 2, 7, 12, A549 cells treated with 11 μM cisplatin; lanes 3, 8, 13, A549rCDDP^2000^ untreated; lanes 4, 9, 14, A549rCDDP^2000^ treated with 11 μM cisplatin; lanes 5, 10, 15, A549rCDDP^2000^ treated with 34 μM cisplatin.
(TIF)

S11 Fig Western blot pATM. Western Blot of pATM in A549 and A549rCDDP^2000^ cells. Lanes 1, 6, 11,: A549 untreated; lanes 2, 7, 12, A549 cells treated with 11 μM cisplatin; lanes 3, 8, 13, A549rCDDP^2000^ untreated; lanes 4, 9, 14, A549rCDDP^2000^ treated with 11 μM cisplatin; lanes 5, 10, 15, A549rCDDP^2000^ treated with 34 μM cisplatin.
(TIF)

S12 Fig Western blot MDM2. Western Blot of MDM2 in A549 (caption in black letters) and A549rCDDP2000 (caption in red letters) cells showing lanes of A549 untreated (caption ctrl), A549 treated with 11 μM cisplatin (caption 11), A549rCDDP2000 untreated (caption ctrl), A549rCDDP2000 treated with 11 μM cisplatin (caption 11) and A549rCDDP2000 treated with 34 μM cisplatin (caption 34). Bands of MDM2 and GAPDH are labelled accordingly.
(TIF)

S13 Fig Western blot p21. Western Blot of p21 in A549 and A549rCDDP^2000^ cells. Lanes 1, 6, 11,: A549 untreated; lanes 2, 7, 12, A549 cells treated with 11 μM cisplatin; lanes 3, 8, 13, A549rCDDP^2000^ untreated; lanes 4, 9, 14, A549rCDDP^2000^ treated with 11 μM cisplatin; lanes 5, 10, 15, A549rCDDP^2000^ treated with 34 μM cisplatin.
(TIF)

S14 Fig Western blot SIP. Western Blot of SIP in A549 (caption in black letters) and A549rCDDP2000 (caption in red letters) cells showing lanes of A549 untreated (caption ctrl), A549 treated with 11 μM cisplatin (caption 11), A549rCDDP2000 untreated (caption ctrl), A549rCDDP2000 treated with 11 μM cisplatin (caption 11) and A549rCDDP2000 treated with 34 μM cisplatin (caption 34). Bands of SIP and GAPDH are labelled accordingly.
(TIF)

S15 Fig Western blot XPC. Western Blot of XPC in A549 (caption in black letters) and A549rCDDP2000 (caption in red letters) cells showing lanes of A549 untreated (caption ctrl), A549 treated with 11 μM cisplatin (caption 11), A549rCDDP2000 untreated (caption ctrl), A549rCDDP2000 treated with 11 μM cisplatin (caption 11) and A549rCDDP2000 treated with 34 μM cisplatin (caption 34). Bands of XPC and GAPDH are labelled accordingly.
(TIF)

S16 Fig Western blot GADD45a. Western Blot of GADD45a in A549 (caption in black letters) and A549rCDDP2000 (caption in red letters) cells showing lanes of A549 untreated (caption ctrl), A549 treated with 11 μM cisplatin (caption 11), A549rCDDP2000 untreated (caption ctrl), A549rCDDP2000 treated with 11 μM cisplatin (caption 11) and A549rCDDP2000 treated with 34 μM cisplatin (caption 34).
(TIF)

S17 Fig Western blot of GAPDH for GADD45a normalization. Western Blot of GAPDH for GADD45a normalization in A549 (caption in black letters) and A549rCDDP^2000^ (caption in red letters) cells showing lanes of A549 untreated (caption ctrl), A549 treated with 11 μM cisplatin (caption 11), A549rCDDP^2000^ untreated (caption ctrl), A549rCDDP^2000^ treated with 11 μM cisplatin (caption 11) and A549rCDDP^2000^ treated with 34 μM cisplatin (caption 34).
(TIF)
