# Supplementary figures and images for: Cisplatin resistance in non-small cell lung cancer cells is associated with an abrogation of cisplatin-induced G2/M cell cycle arrest
Source: PLoS One. 2017 Jul 26;12(7):e0181081. doi: 10.1371/journal.pone.0181081 (PMC5528889; doi:10.1371/journal.pone.0181081)

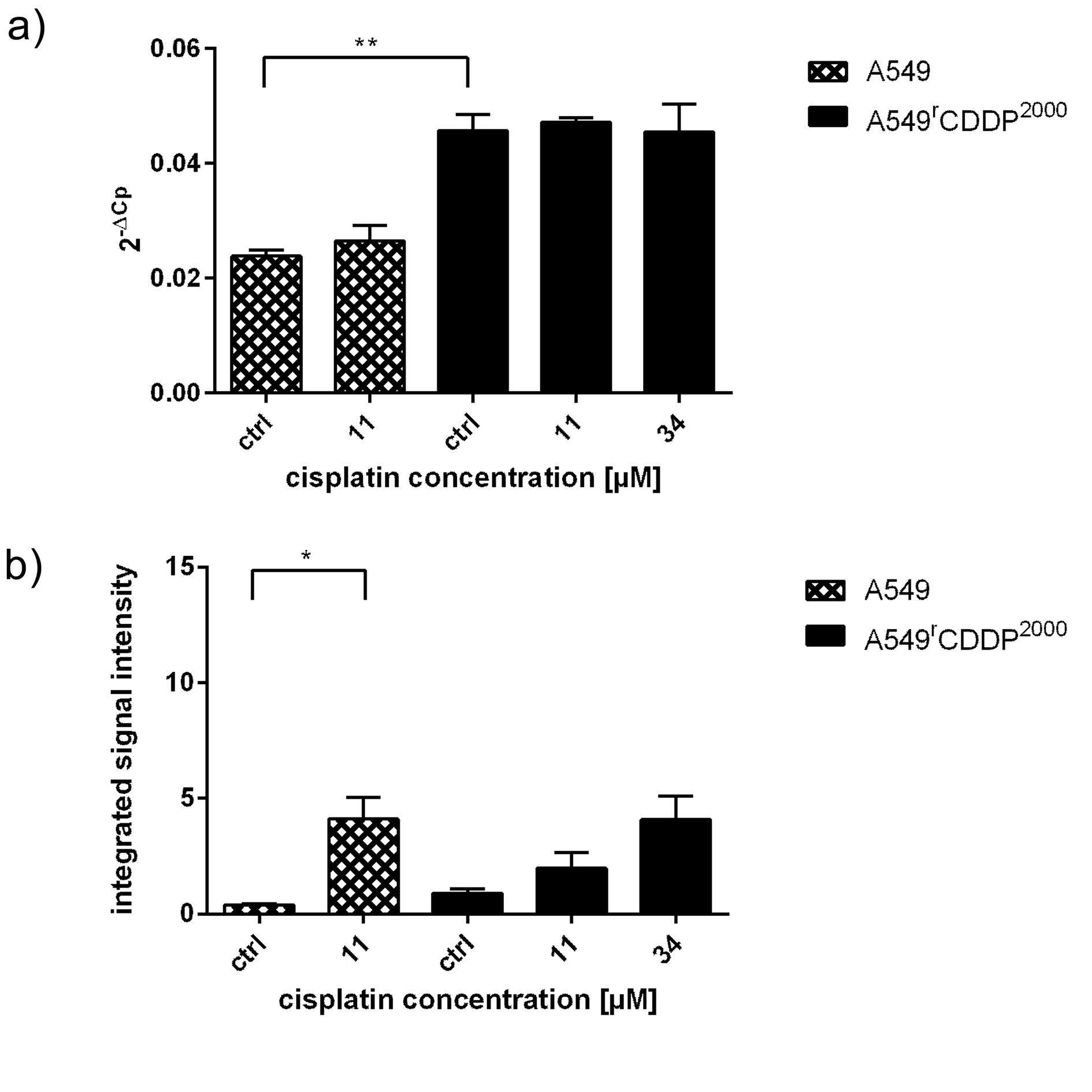

Supplement: S1 Fig — Analysis of p53 in a) RT-PCR (n = 3) as individual data points presented as mean ± SEM and b) Western Blot (n = 3) as integrated signal intensity normalized to the housekeeper α-actin in A549 and A549rCDDP2000 cells, presented as mean ± SEM. (TIF) [file pone.0181081.s001.tif]

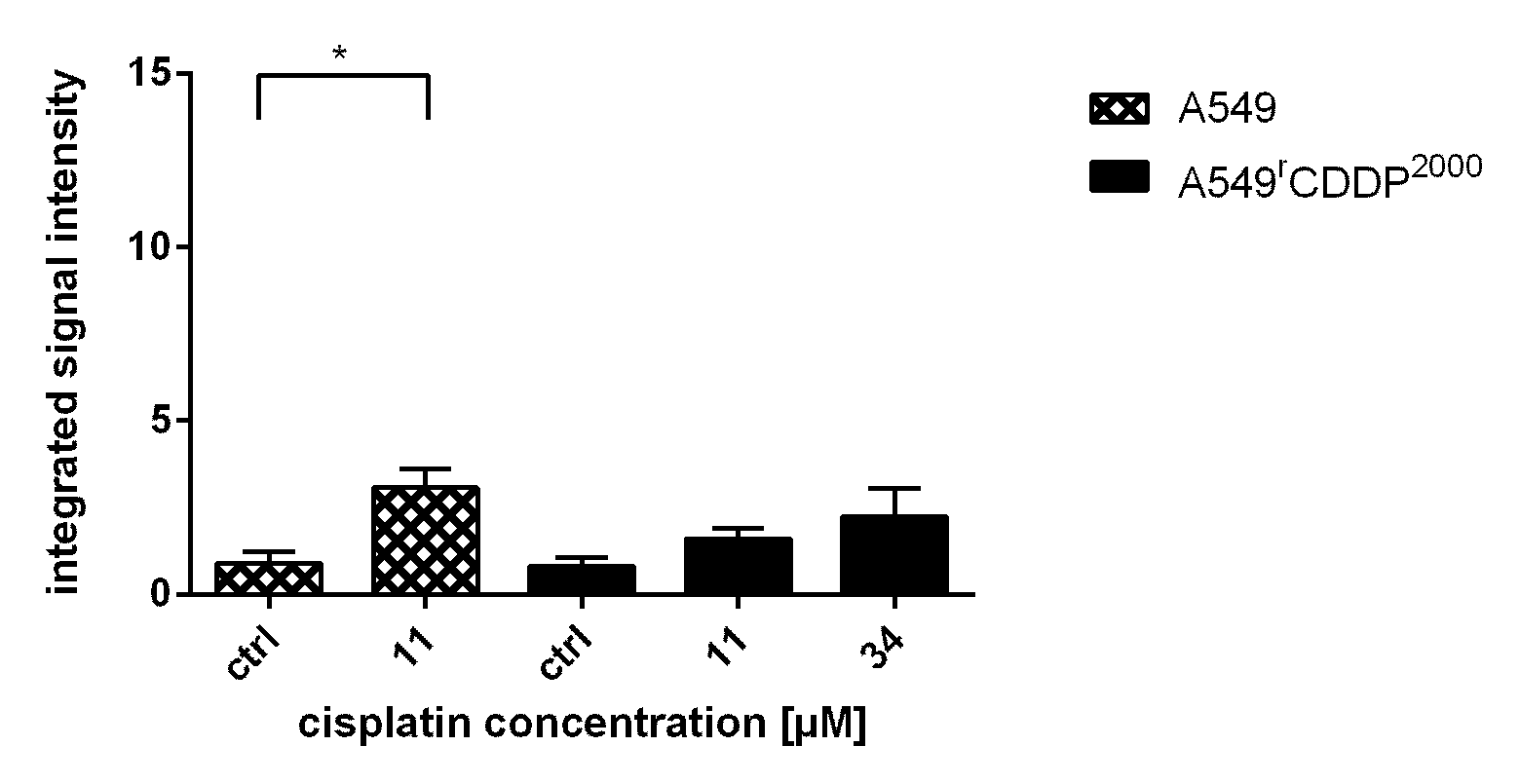

Supplement: S2 Fig — Western Blot Analysis of pAtm (n = 3) a) as integrated signal intensity normalized to the housekeeper α-actin in A549 and A549rCDDP2000 cells, presented as mean ± SEM. (TIF) [file pone.0181081.s002.tif]

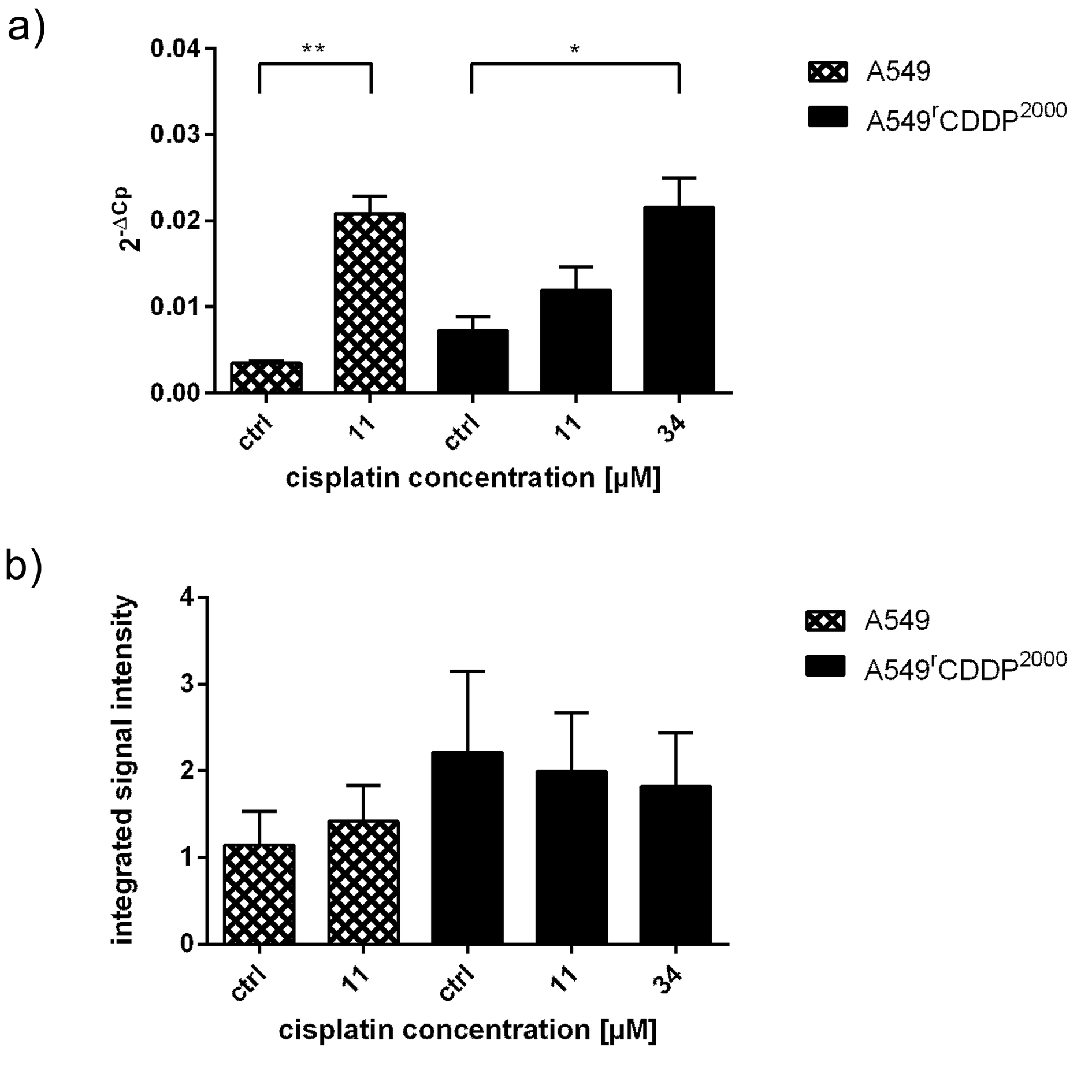

Supplement: S3 Fig — Analysis of MDM2 in a) RT-PCR (n = 3) as individual data points presented as mean ± SEM and b) Western Blot (n = 3) as integrated signal intensity normalized to the housekeeper GAPDH in A549 and A549rCDDP2000 cells, presented as mean ± SEM. (TIF) [file pone.0181081.s003.tif]

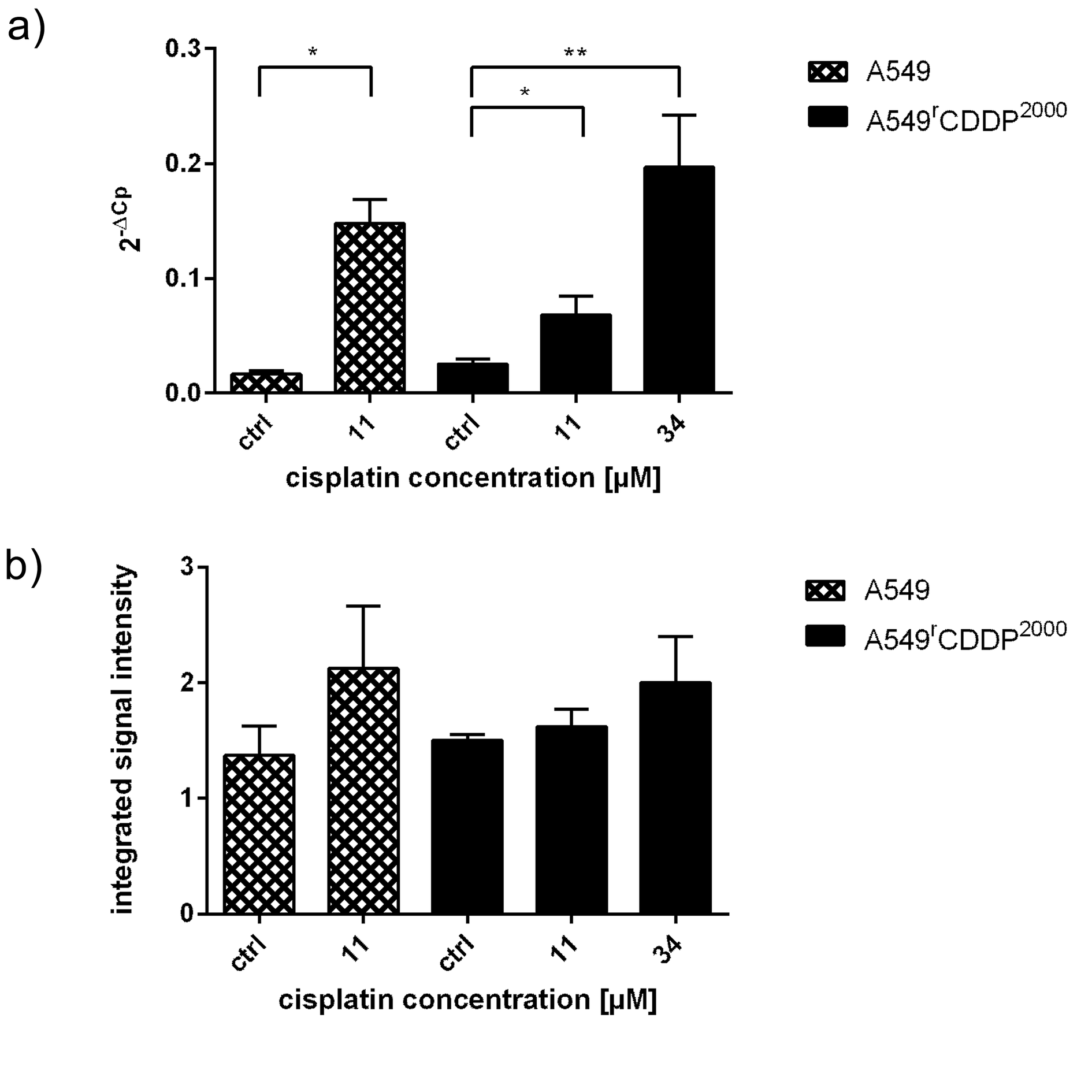

Supplement: S4 Fig — Analysis of p21 in a) RT-PCR (n = 3) as individual data points presented as mean ± SEM and b) Western Blot (n = 3) as integrated signal intensity normalized to the housekeeper α-actin in A549 and A549rCDDP2000 cells, presented as mean ± SEM. (TIF) [file pone.0181081.s004.tif]

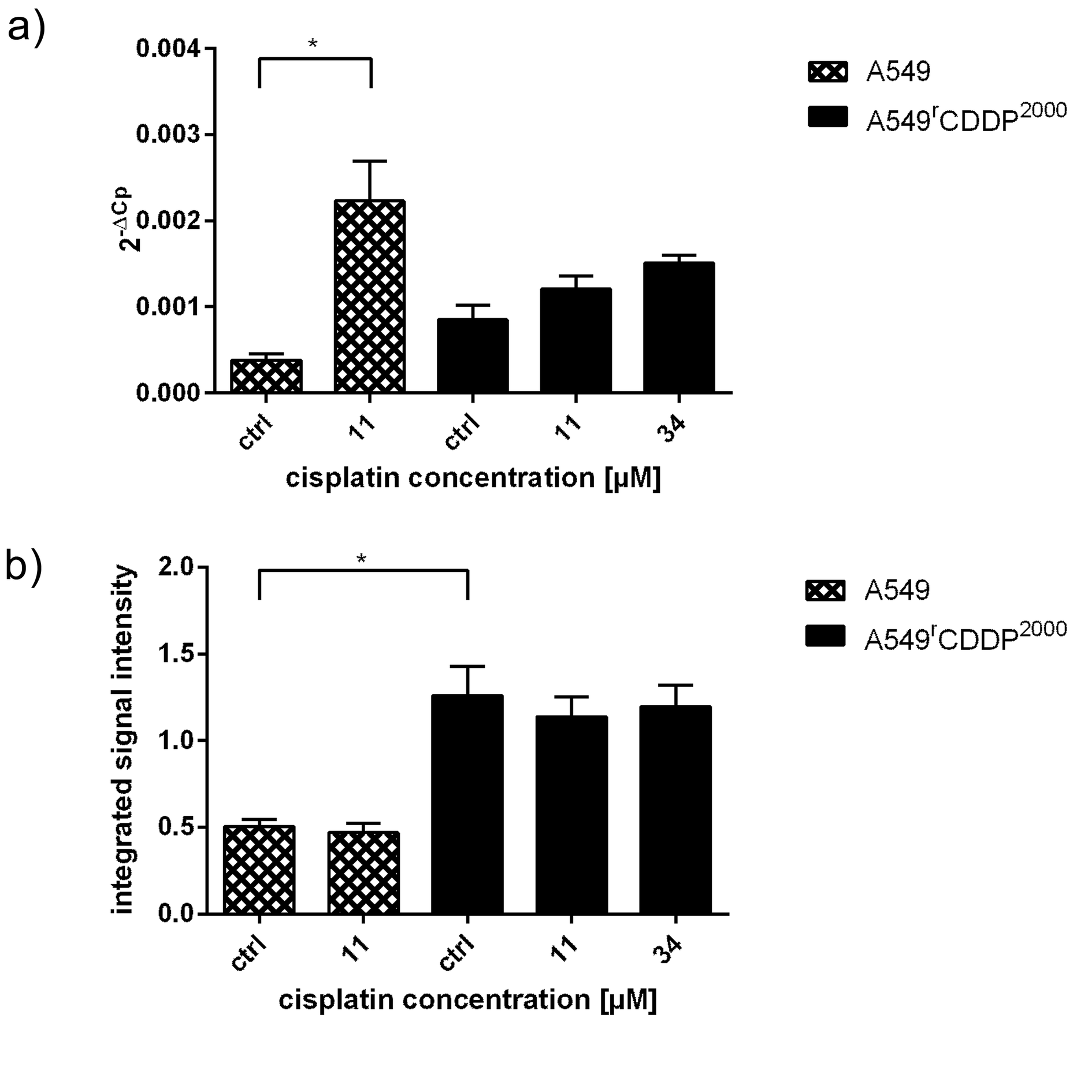

Supplement: S5 Fig — Analysis of SIP in a) RT-PCR (n = 3) as individual data points presented as mean ± SEM and b) Western Blot (n = 3) as integrated signal intensity normalized to the housekeeper GAPDH in A549 and A549rCDDP2000 cells, presented as mean ± SEM. (TIF) [file pone.0181081.s005.tif]

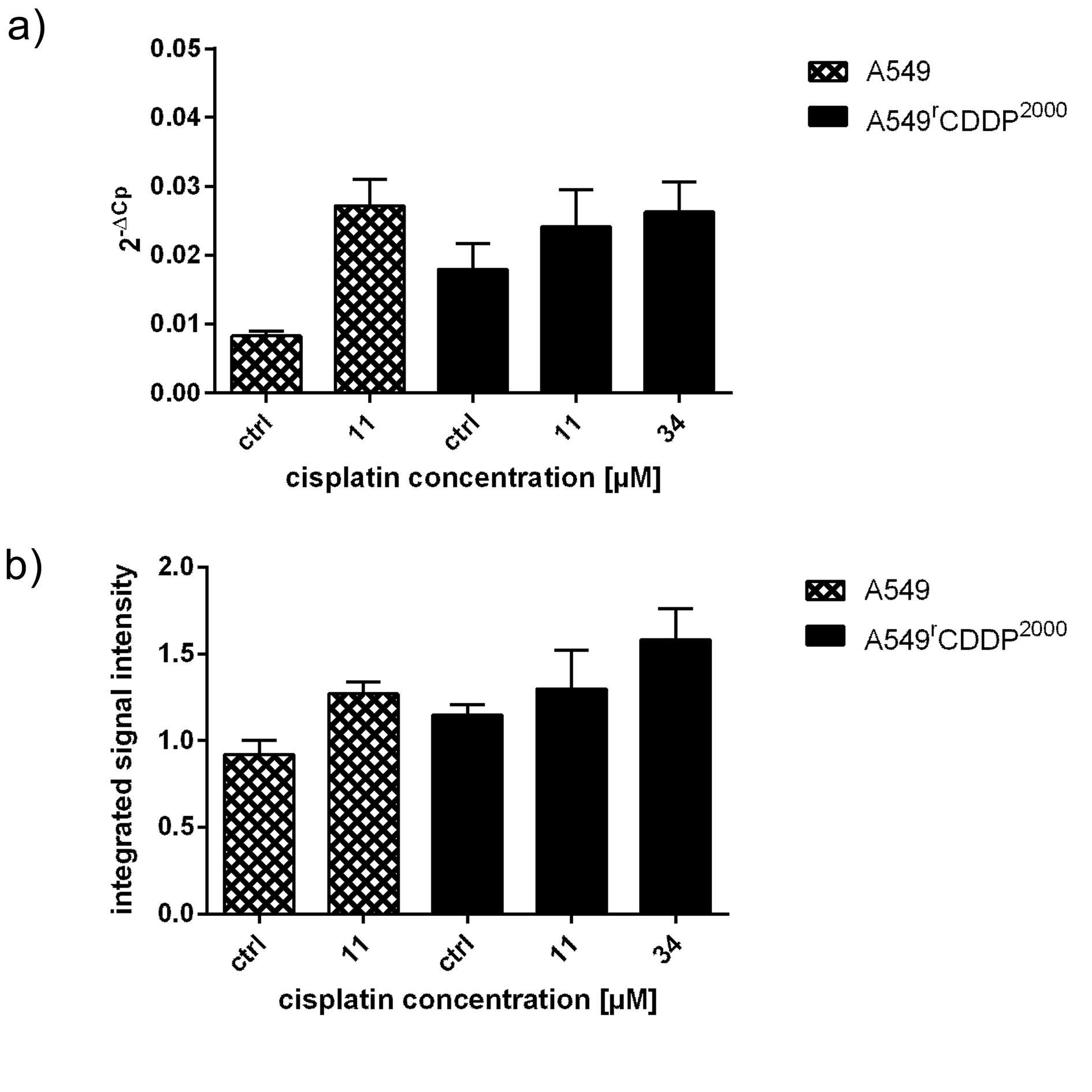

Supplement: S6 Fig — Analysis of XPC in a) RT-PCR (n = 3) as individual data points presented as mean ± SEM and b) Western Blot (n = 3) as integrated signal intensity normalized to the housekeeper GAPDH in A549 and A549rCDDP2000 cells, presented as mean ± SEM. (TIF) [file pone.0181081.s006.tif]

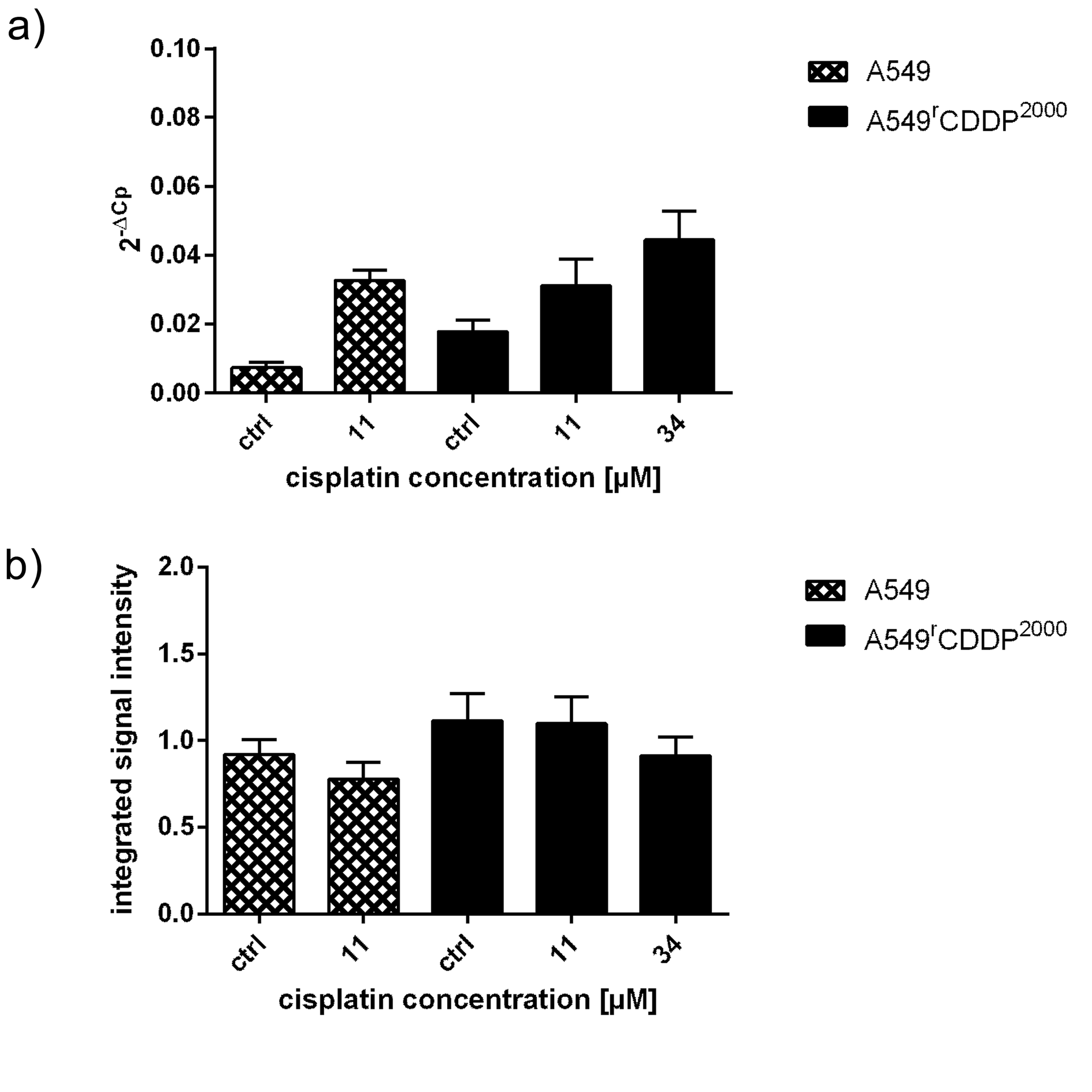

Supplement: S7 Fig — Analysis of GADD45a in a) RT-PCR (n = 3) as individual data points presented as mean ± SEM and b) Western Blot (n = 3) as integrated signal intensity normalized to the housekeeper GAPDH in A549 and A549rCDDP2000 cells, presented as mean ± SEM. (TIF) [file pone.0181081.s007.tif]

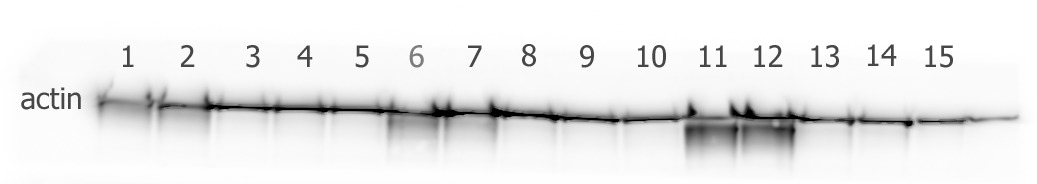

Supplement: S9 Fig — Western Blot of α-Actin in A549 and A549rCDDP2000 cells. Lanes 1, 6, 11,: A549 untreated; lanes 2, 7, 12, A549 cells treated with 11 μM cisplatin; lanes 3, 8, 13, A549rCDDP2000 untreated; lanes 4, 9, 14, A549rCDDP2000 treated with 11 μM cisplatin; lanes 5, 10, 15, A549rCDDP2000 treated with 34 μM cisplatin. (TIF) [file pone.0181081.s009.tif]

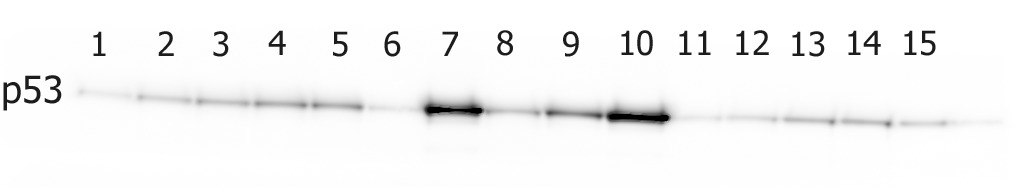

Supplement: S10 Fig — Western Blot of p53 in A549 and A549rCDDP2000 cells. Lanes 1, 6, 11,: A549 untreated; lanes 2, 7, 12, A549 cells treated with 11 μM cisplatin; lanes 3, 8, 13, A549rCDDP2000 untreated; lanes 4, 9, 14, A549rCDDP2000 treated with 11 μM cisplatin; lanes 5, 10, 15, A549rCDDP2000 treated with 34 μM cisplatin. (TIF) [file pone.0181081.s010.tif]

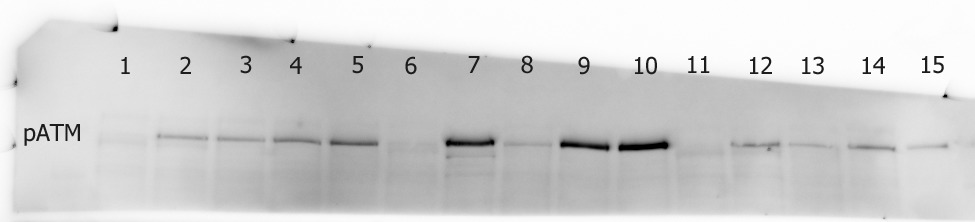

Supplement: S11 Fig — Western Blot of pATM in A549 and A549rCDDP2000 cells. Lanes 1, 6, 11,: A549 untreated; lanes 2, 7, 12, A549 cells treated with 11 μM cisplatin; lanes 3, 8, 13, A549rCDDP2000 untreated; lanes 4, 9, 14, A549rCDDP2000 treated with 11 μM cisplatin; lanes 5, 10, 15, A549rCDDP2000 treated with 34 μM cisplatin. (TIF) [file pone.0181081.s011.tif]

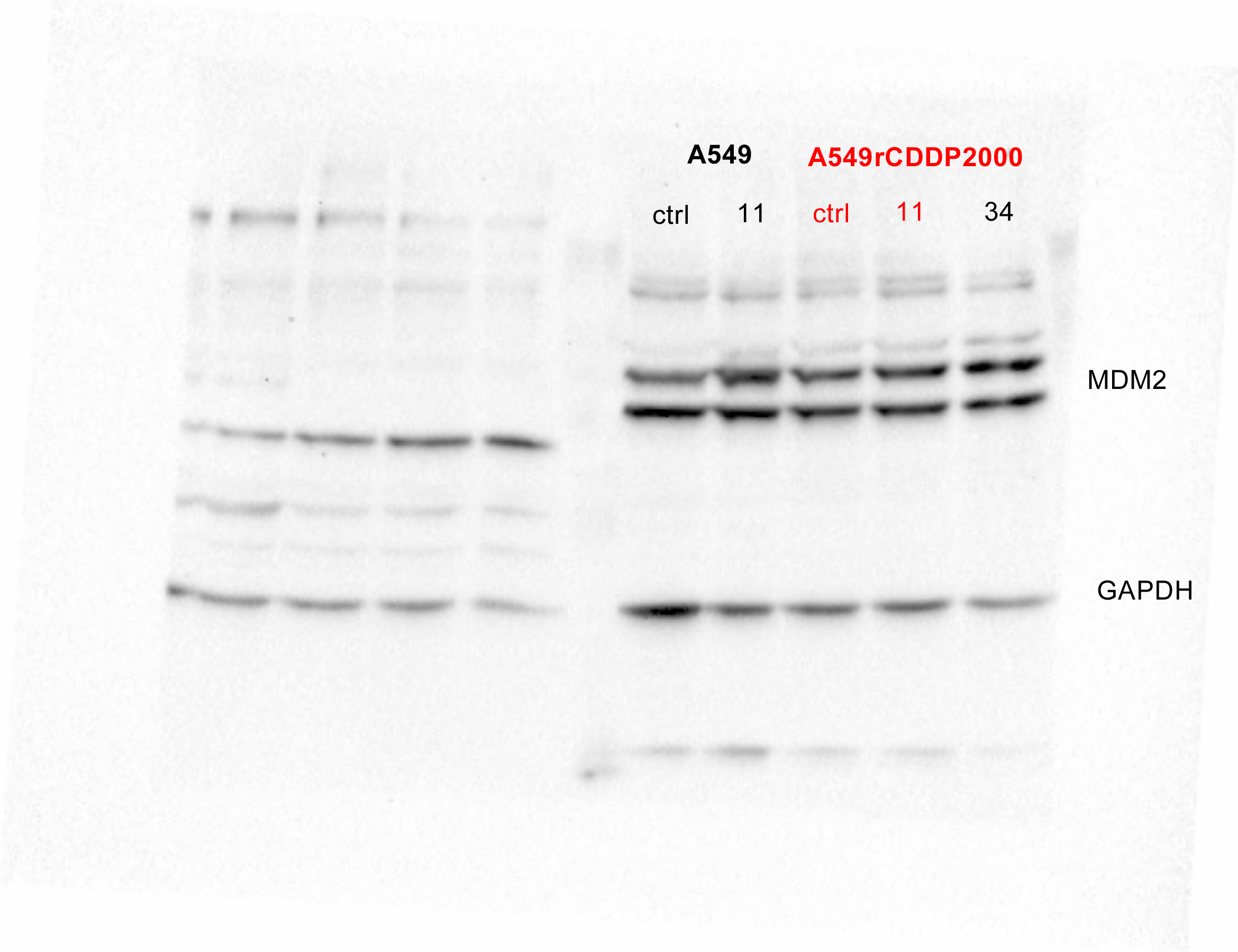

Supplement: S12 Fig — Western Blot of MDM2 in A549 (caption in black letters) and A549rCDDP2000 (caption in red letters) cells showing lanes of A549 untreated (caption ctrl), A549 treated with 11 μM cisplatin (caption 11), A549rCDDP2000 untreated (caption ctrl), A549rCDDP2000 treated with 11 μM cisplatin (caption 11) and A549rCDDP2000 treated with 34 μM cisplatin (caption 34). Bands of MDM2 and GAPDH are labelled accordingly. (TIF) [file pone.0181081.s012.tif]

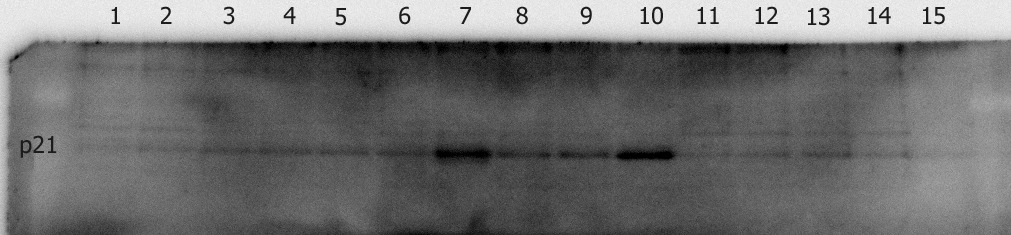

Supplement: S13 Fig — Western Blot of p21 in A549 and A549rCDDP2000 cells. Lanes 1, 6, 11,: A549 untreated; lanes 2, 7, 12, A549 cells treated with 11 μM cisplatin; lanes 3, 8, 13, A549rCDDP2000 untreated; lanes 4, 9, 14, A549rCDDP2000 treated with 11 μM cisplatin; lanes 5, 10, 15, A549rCDDP2000 treated with 34 μM cisplatin. (TIF) [file pone.0181081.s013.tif]

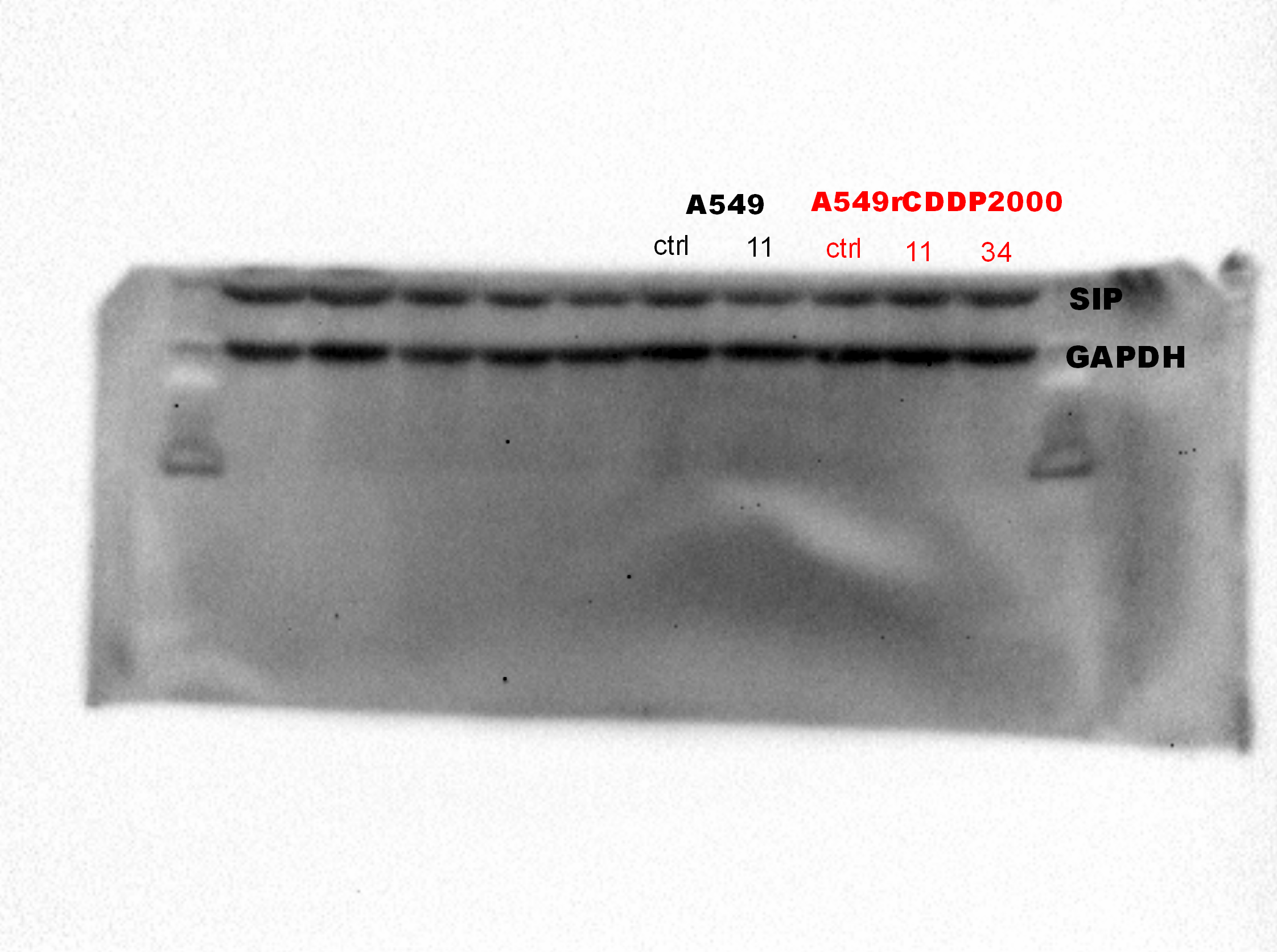

Supplement: S14 Fig — Western Blot of SIP in A549 (caption in black letters) and A549rCDDP2000 (caption in red letters) cells showing lanes of A549 untreated (caption ctrl), A549 treated with 11 μM cisplatin (caption 11), A549rCDDP2000 untreated (caption ctrl), A549rCDDP2000 treated with 11 μM cisplatin (caption 11) and A549rCDDP2000 treated with 34 μM cisplatin (caption 34). Bands of SIP and GAPDH are labelled accordingly. (TIF) [file pone.0181081.s014.tif]

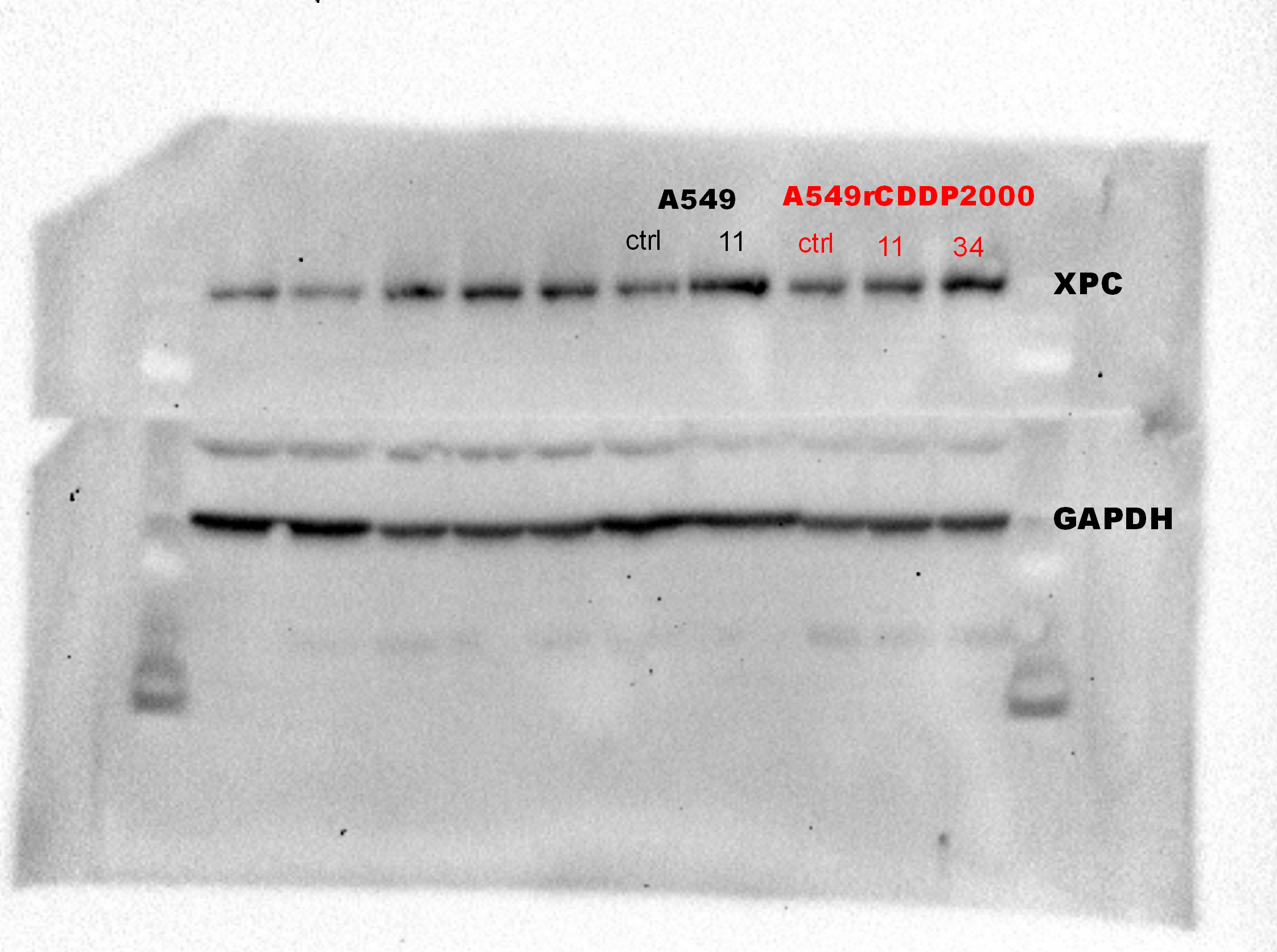

Supplement: S15 Fig — Western Blot of XPC in A549 (caption in black letters) and A549rCDDP2000 (caption in red letters) cells showing lanes of A549 untreated (caption ctrl), A549 treated with 11 μM cisplatin (caption 11), A549rCDDP2000 untreated (caption ctrl), A549rCDDP2000 treated with 11 μM cisplatin (caption 11) and A549rCDDP2000 treated with 34 μM cisplatin (caption 34). Bands of XPC and GAPDH are labelled accordingly. (TIF) [file pone.0181081.s015.tif]

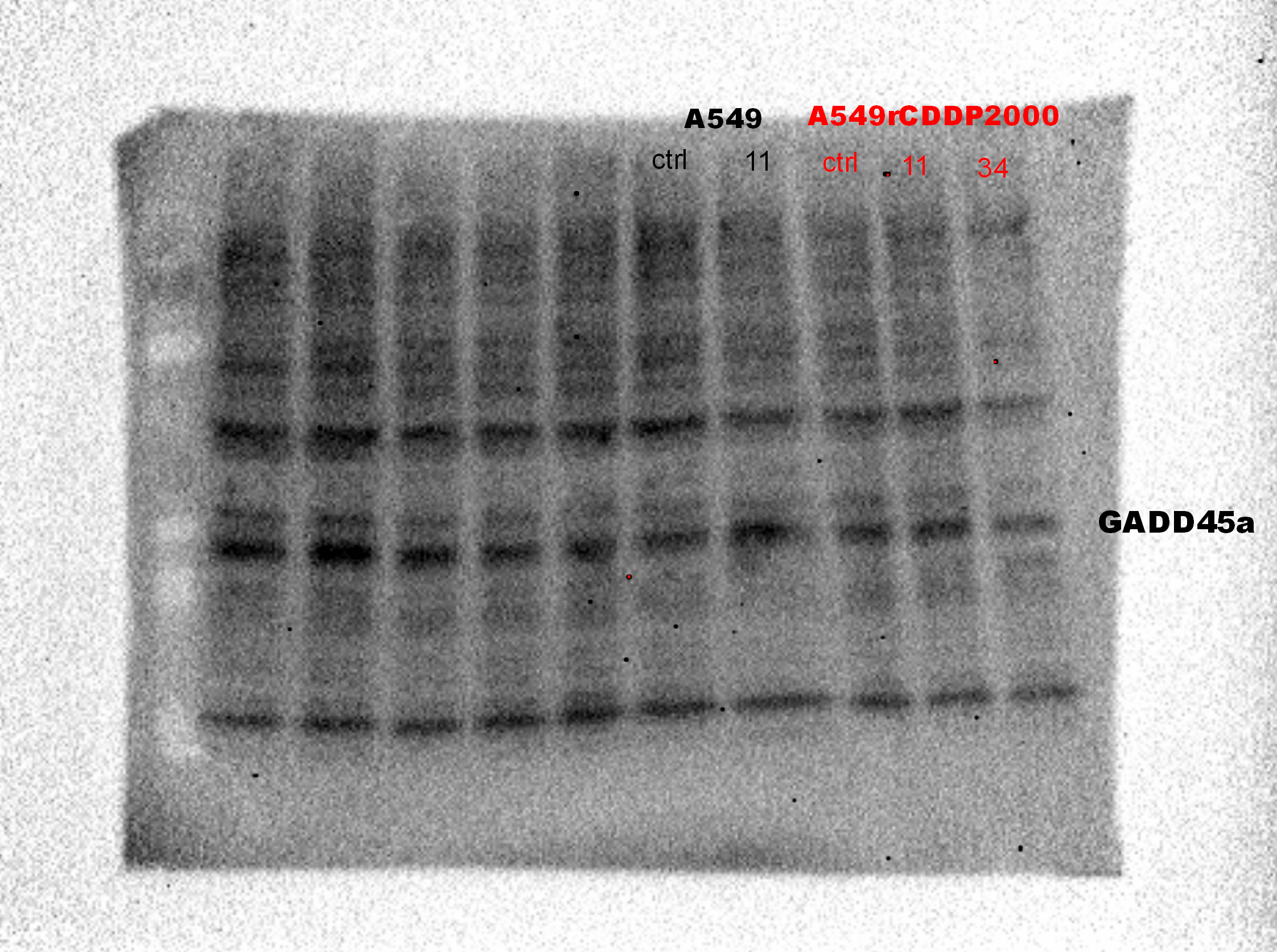

Supplement: S16 Fig — Western Blot of GADD45a in A549 (caption in black letters) and A549rCDDP2000 (caption in red letters) cells showing lanes of A549 untreated (caption ctrl), A549 treated with 11 μM cisplatin (caption 11), A549rCDDP2000 untreated (caption ctrl), A549rCDDP2000 treated with 11 μM cisplatin (caption 11) and A549rCDDP2000 treated with 34 μM cisplatin (caption 34). (TIF) [file pone.0181081.s016.tif]

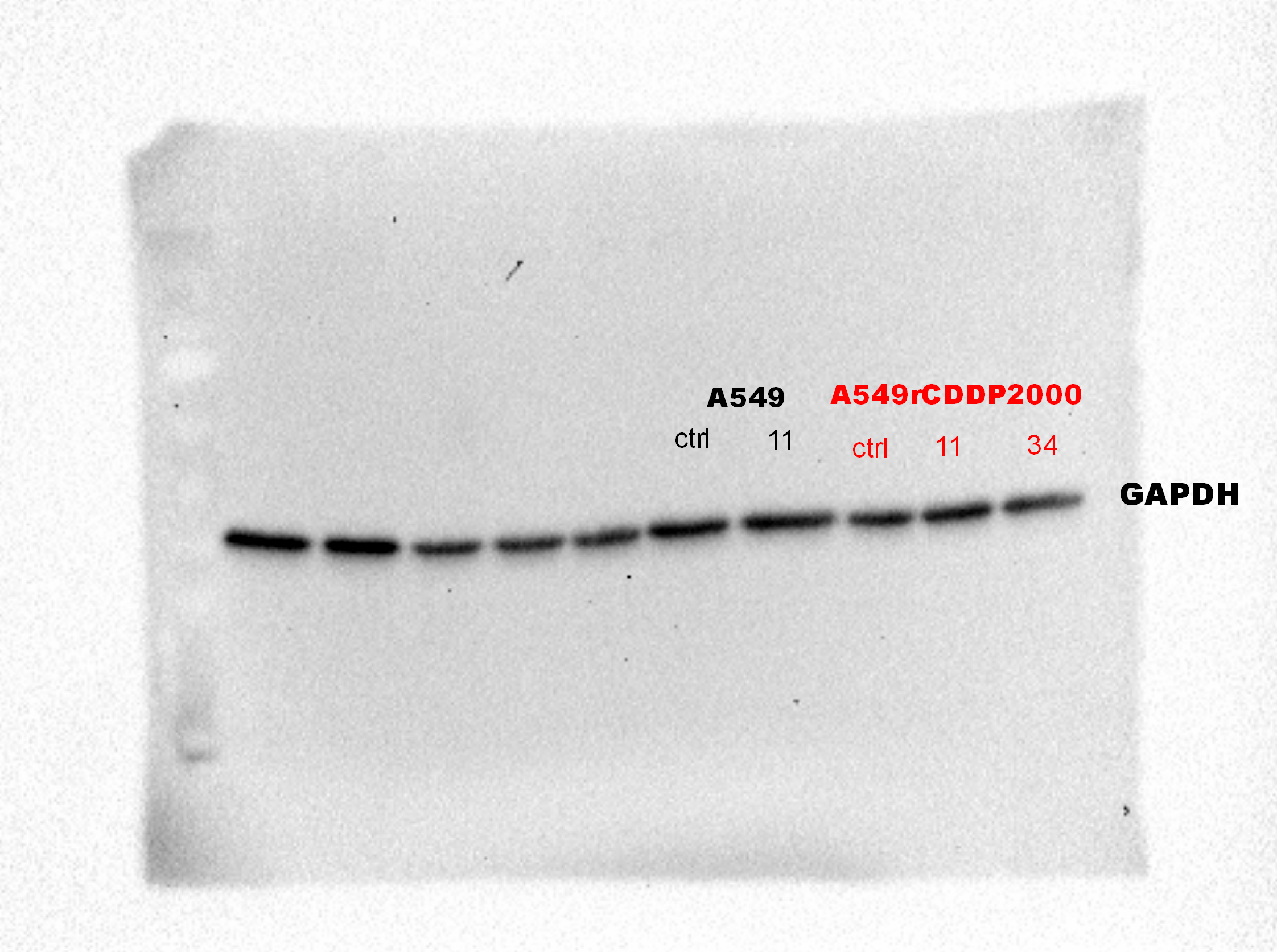

Supplement: S17 Fig — Western Blot of GAPDH for GADD45a normalization in A549 (caption in black letters) and A549rCDDP2000 (caption in red letters) cells showing lanes of A549 untreated (caption ctrl), A549 treated with 11 μM cisplatin (caption 11), A549rCDDP2000 untreated (caption ctrl), A549rCDDP2000 treated with 11 μM cisplatin (caption 11) and A549rCDDP2000 treated with 34 μM cisplatin (caption 34). (TIF) [file pone.0181081.s017.tif]
