# Supplementary material for: Cisplatin resistance in non-small cell lung cancer cells is associated with an abrogation of cisplatin-induced G2/M cell cycle arrest
Source: PLoS One. 2017 Jul 26;12(7):e0181081. doi: 10.1371/journal.pone.0181081 (PMC5528889; doi:10.1371/journal.pone.0181081)
Supplement: S8 Fig — Tables with individual data values analysed for preparation of all figures in this manuscript. (DOCX) [file pone.0181081.s008.docx]

**Fig 1 Cytotoxicity (MTT)**

Values of the representative sigmoidal concentration-response curve of cisplatin in A549 and A549^r^CDDP^2000^ cells. Survival is expressed in terms of % of absorbance of untreated cells as mean ± SD.

|  | A549 | | | | A549^r^CDDP^2000^ | | | |
| --- | --- | --- | --- | --- | --- | --- | --- | --- |
|  | Absorption [%] | | Absorption [units] | | Absorption [%] | | Absorption [units] | |
| log [conc.] | Mean | SD | Mean | SD | Mean | SD | Mean | SD |
| control | 100.0 | 4.1 | 0.508 | 0.021 | 100.0 | 1.4 | 0.464 | 0.007 |
| -6.301 | 107.2 | 8.6 | 0.544 | 0.044 | 91.2 | 1.1 | 0.423 | 0.005 |
| -6.000 | 101.5 | 3.0 | 0.516 | 0.015 | 100.2 | 3.1 | 0.465 | 0.014 |
| -5.301 | 107.7 | 3.8 | 0.547 | 0.019 | 98.3 | 4.1 | 0.456 | 0.019 |
| -5.000 | 97.2 | 3.6 | 0.494 | 0.018 | 98.3 | 11.0 | 0.456 | 0.051 |
| -4.523 | 40.4 | 3.9 | 0.205 | 0.020 | 89.4 | 3.1 | 0.414 | 0.014 |
| -4.301 | 24.7 | 4.4 | 0.125 | 0.023 | 63.8 | 6.4 | 0.296 | 0.030 |
| -4.155 | 23.4 | 2.5 | 0.119 | 0.012 | 37.0 | 5.2 | 0.171 | 0.024 |
| -4.000 | 20.5 | 2.2 | 0.104 | 0.011 | 16.8 | 1.4 | 0.078 | 0.007 |
| -3.301 | 12.1 | 0.4 | 0.061 | 0.002 | 9.4 | 0.9 | 0.044 | 0.004 |

Sensitivity of A549 and A549^r^CDDP^2000^ cells towards cisplatin expressed as pEC50 (results of single testing days, n = 11-12).

| Cell line | A549 | A549^r^CDDP^2000^ |
| --- | --- | --- |
| pEC_50_ | -4.744 | -4.667 |
|  | -4.554 | -4.392 |
|  | -4.537 | -4.234 |
|  | -4.671 | -4.261 |
|  | -4.248 | -4.024 |
|  | -4.329 | -4.093 |
|  | -4.662 | -4.066 |
|  | -4.500 | -4.349 |
|  | -4.487 | -4.307 |
|  | -4.511 | -4.266 |
|  | -4.498 | -4.288 |
|  |  | -4.196 |
| Mean (SD) | -4.522 (0.144) | -4.626 (0.171) |

**Fig 2 Intracellular platinum content**

Cellular platinum accumulation in A549 and A549^r^CDDP^2000^ cells, treated with 11 μM or 34 μM cisplatin (results of single testing days, n = 29-33).

| Cell line | A549 | | A549^r^CDDP^2000^ |
| --- | --- | --- | --- |
| Treatment concentration | 11 µM | 11 µM | 34 µM |
| Cellular platinum accumulation | 0.108 | 0.083 | 0.212 |
| [µmol platinum/g protein] | 0.099 | 0.074 | 0.239 |
|  | 0.108 | 0.105 | 0.257 |
|  | 0.119 | 0.079 | 0.134 |
|  | 0.103 | 0.057 | 0.128 |
|  | 0.110 | 0.049 | 0.206 |
|  | 0.083 | 0.059 | 0.198 |
|  | 0.074 | 0.079 | 0.212 |
|  | 0.078 | 0.073 | 0.207 |
|  | 0.096 | 0.076 | 0.230 |
|  | 0.087 | 0.046 | 0.156 |
|  | 0.092 | 0.080 | 0.233 |
|  | 0.062 | 0.061 | 0.250 |
|  | 0.074 | 0.066 | 0.271 |
|  | 0.071 | 0.064 | 0.104 |
|  | 0.072 | 0.054 | 0.198 |
|  | 0.100 | 0.035 | 0.217 |
|  | 0.096 | 0.039 | 0.174 |
|  | 0.030 | 0.042 | 0.155 |
|  | 0.038 | 0.038 | 0.179 |
|  | 0.050 | 0.039 | 0.067 |
|  | 0.041 | 0.043 | 0.070 |
|  | 0.052 | 0.025 | 0.085 |
|  | 0.053 | 0.019 | 0.075 |
|  | 0.026 | 0.037 | 0.068 |
|  | 0.029 | 0.025 | 0.066 |
|  | 0.031 | 0.030 | 0.068 |
|  | 0.029 | 0.016 | 0.059 |
|  | 0.034 | 0.023 | 0.072 |
|  | 0.037 | 0.023 |  |
|  | 0.030 | 0.034 |  |
|  | 0.034 |  |  |
|  | 0.036 |  |  |
| Mean (SEM) | 0.066 (0.005) | 0.051 (0.004) | 0.158 (0.013) |

**Fig 3 Cisplatin-DNA adduct formation**

Cisplatin-DNA adduct formation in A549 and A549^r^CDDP^2000^ cells, treated with 11 μM or 34 μM cisplatin for 4h and 24h with densitometric values (results of single testing days, n = 3).

| Cell line | A549 | | | A549^r^CDDP^2000^ | | |
| --- | --- | --- | --- | --- | --- | --- |
| Treatment duration | 4 h | | | 24 h | | |
| Treatment concentration | 11 µM | 11 µM | 34 µM | 11 µM | 11 µM | 34 µM |
|  | 12.74 | 6.83 | 20.12 | 6.75 | 3.45 | 7.55 |
|  | 13.91 | 8.56 | 11.39 | 30.87 | 12.13 | 23.14 |
|  | 12.65 | 10.81 | 25.60 | 22.12 | 8.04 | 20.78 |
| Mean (SEM) | 13.10 (0.41) | 8.73 (1.15) | 19.04 (4.14) | 19.91 (7.05) | 7.87 (2.51) | 17.16 (4.85) |

**Fig 4 Cell cycle analysis**

Cell cycle analysis of cell fraction in % in G_1_/G_0_-phase, S-phase and G_2_/M-phase in A549 and A549^r^CDDP^2000^ cells (results of single testing days, n = 3-5).

| Cell line | A549 | | A549^r^CDDP^2000^ | | |
| --- | --- | --- | --- | --- | --- |
| Treatment concentration | control | 11 µM | control | 11 µM | 34 µM |
| Cell phase | Cell fraction [%] | Cell fraction [%] | Cell fraction [%] | Cell fraction [%] | Cell fraction [%] |
| G_1_/G_0_ | 70.87 | 25.82 | 67.46 | 40.46 | 47.78 |
|  | 72.30 | 18.59 | 66.79 | 50.97 | 47.58 |
|  | 69.25 | 11.87 | 72.82 | 58.25 | 52.56 |
|  | 68.38 | 11.68 |  |  | 50.54 |
|  | 75.82 | 50.13 |  |  | 50.52 |
|  | 75.89 | 47.38 |  |  | 51.94 |
| Mean (SEM) | 72.09 (1.31) | 27.58 (7.03) | 69.02 (1.91) | 49.89 (5.16) | 50.15 (0.85) |
| S | 11.39 | 9.87 | 9.86 | 27.54 | 10.77 |
|  | 7.81 | 8.66 | 13.35 | 21.43 | 16.07 |
|  | 12.13 | 10.21 | 10.70 | 17.67 | 15.50 |
|  | 12.50 | 6.68 |  |  | 13.23 |
|  | 8.39 | 22.23 |  |  | 19.96 |
|  | 9.92 | 24.91 |  |  | 15.98 |
| Mean (SEM) | 10.36 (0.80) | 13.76 (3.16) | 11.30 (1.05) | 22.21 (2.88) | 15.25 (1.26) |
| G_2_/M | 14.12 | 44.50 | 11.07 | 11.17 | 4.56 |
|  | 15.36 | 54.99 | 10.69 | 10.43 | 4.13 |
|  | 15.80 | 62.10 | 5.47 | 9.04 | 6.55 |
|  | 16.61 | 62.74 |  |  | 6.26 |
|  | 11.94 | 11.81 |  |  | 5.28 |
|  | 11.14 | 12.93 |  |  | 6.45 |
| Mean (SEM) | 14.16 (0.90) | 41.51 (9.60) | 9.08 (1.81) | 10.21 (0.62) | 5.54 (0.42) |

**Fig 5 Apoptosis induction**

Apoptosis analysis with FITC Annexin (n = 3-4) and cell count in SubG_1_-phase (n = 3-6) as fold change related to untreated controls in A549 and A549^r^CDDP^2000^ cells.

| Cell line | A549 | A549^r^CDDP^2000^ | |
| --- | --- | --- | --- |
| Treatment concentration | 11 µM | 11 µM | 34 µM |
| FITC-Annexin [fold change to untreated control] | | | |
|  | 6.922 | 2.141 | 4.956 |
|  | 4.994 | 2.114 | 2.811 |
|  | 5.649 | 1.027 | 3.374 |
|  |  | 2.516 |  |
| Mean (SEM) | 5.856 (0.566) | 1.950 (0.321) | 3.714 (0.642) |
| SubG_1_-phase [fold change to untreated control] | | | |
|  | 5.470 | 1.948 | 3.347 |
|  | 4.190 | 1.672 | 3.048 |
|  | 5.296 | 1.380 | 2.288 |
|  | 7.112 |  |  |
|  | 3.973 |  |  |
|  | 4.599 |  |  |
| Mean (SEM) | 5.107 (0.468) | 1.667 (0.164) | 2.894 (0.315) |

**Fig 6 and S_Fig 1 p53**

Results of p53 in real time RT-PCR (n = 3) as fold change relative to untreated control using the ΔΔCp-method and individual data points calculated using the ΔCp-method, and densitometric protein results in Western blot (n = 3) as fold change and integrated signal intensity; normalised to the housekeeper α-actin in A549 and A549^r^CDDP^2000^ cells.

| Cell line | A549 | | A549^r^CDDP^2000^ | | |
| --- | --- | --- | --- | --- | --- |
| Treatment concentration | control | 11 µM | control | 11 µM | 34 µM |
| mRNA (fold change relative to untreated control) | | | | | |
|  |  | 1.015 |  | 1.140 | 0.979 |
|  |  | 1.438 |  | 0.952 | 0.806 |
|  |  | 0.907 |  | 1.024 | 1.227 |
| Mean (SEM) |  | 1.120 (0.162) |  | 1.038 (0.055) | 1.004 (0.122) |
| mRNA (individual data points) | | | | | |
|  | 0.024 | 0.024 | 0.041 | 0.047 | 0.040 |
|  | 0.022 | 0.032 | 0.051 | 0.048 | 0.041 |
|  | 0.026 | 0.023 | 0.045 | 0.046 | 0.055 |
| Mean (SEM) | 0.024 (0.001) | 0.026 (0.003) | 0.046 (0.003) | 0.047 (0.001) | 0.045 (0.005) |
| Protein (fold change relative to untreated control) | | | | | |
|  |  | 11.44 |  | 2.32 | 6.64 |
|  |  | 11.05 |  | 2.55 | 3.19 |
|  |  | 9.24 |  | 1.37 | 4.34 |
| Mean (SEM) |  | 10.58 (0.68) |  | 2.08 (0.36) | 4.72 (1.01) |
| Protein (integrated signal intensity) | | | | | |
|  | 0.48 | 5.54 | 0.90 | 2.08 | 5.95 |
|  | 0.40 | 4.48 | 1.22 | 3.11 | 3.89 |
|  | 0.25 | 2.32 | 0.55 | 0.75 | 2.39 |
| Mean (SEM) | 0.38 (0.07) | 4.11 (0.95) | 0.89 (0.19) | 1.98 (0.68) | 4.01 (1.03) |

**Fig 7 and S_Fig 2 pATM**

Results of pATM as densitometric protein results in Western blot (n = 3) as fold change and integrated signal intensity normalised to the housekeeper α-actin in A549 and A549^r^CDDP^2000^ cells.

| Cell line | A549 | | A549^r^CDDP^2000^ | | |
| --- | --- | --- | --- | --- | --- |
| Treatment concentration | control | 11 µM | control | 11 µM | 34 µM |
| Protein (fold change relative to untreated control) | | | | | |
|  |  | 1.92 |  | 2.04 | 2.87 |
|  |  | 15.35 |  | 3.50 | 2.17 |
|  |  | 2.34 |  | 1.59 | 2.90 |
| Mean (SEM) |  | 6.54 (4.41) |  | 2.38 (0.58) | 2.65 (0.24) |
| Protein (integrated signal intensity) | | | | | |
|  | 1.12 | 2.15 | 0.93 | 1.90 | 2.67 |
|  | 0.26 | 3.99 | 0.30 | 1.05 | 0.65 |
|  | 1.32 | 3.09 | 1.18 | 1.88 | 3.42 |
| Mean (SEM) | 0.90 (0.33) | 3.08 (0.53) | 0.80 (0.26) | 1.61 (0.28) | 2.25 (0.83) |

**Fig 8 and S_Fig 3 MDM2**

Results of MDM2 in real time RT-PCR (n = 3) as fold change relative to untreated control calculated using the ΔΔCp-method and individual data points calculated using the ΔCp-method and densitometric protein results in Western blot (n = 7) as fold change and integrated signal intensity; normalised to the housekeeper α-actin in A549 and A549^r^CDDP^2000^ cells.

| Cell line | A549 | | A549^r^CDDP^2000^ | | |
| --- | --- | --- | --- | --- | --- |
| Treatment concentration | control | 11 µM | control | 11 µM | 34 µM |
| mRNA (fold change relative to untreated control) | | | | | |
|  |  | 4.785 |  | 1.520 | 3.125 |
|  |  | 4.719 |  | 1.535 | 1.973 |
|  |  | 3.998 |  | 1.429 | 2.596 |
| Mean (SEM) |  | 4.501 (0.252) |  | 1.495 (0.033) | 2.565 (0.333) |
| mRNA (individual data points) | | | | | |
|  | 0.003 | 0.020 | 0.007 | 0.011 | 0.027 |
|  | 0.004 | 0.025 | 0.010 | 0.017 | 0.023 |
|  | 0.003 | 0.018 | 0.005 | 0.007 | 0.015 |
| Mean (SEM) | 0.003 (0.0002) | 0.021 (0.002) | 0.007 (0.002) | 0.012 (0.003) | 0.022 (0.003) |
| Protein (fold change relative to untreated control) | | | | | |
|  |  | 2.87 |  | 0.94 | 1.27 |
|  |  | 0.86 |  | 0.91 | 0.83 |
|  |  | 1.76 |  | 1.11 | 1.77 |
|  |  | 0.87 |  | 0.95 | 0.91 |
|  |  | 1.96 |  | 1.50 | 1.16 |
|  |  | 1.01 |  | 1.08 | 0.61 |
|  |  | 0.97 |  | 0.65 | 0.56 |
| Mean (SEM) |  | 1.47 (0.29) |  | 1.02 (0.10) | 1.01 (0.16) |
| Protein (integrated signal intensity) | | | | | |
|  | 0.21 | 0.18 | 0.23 | 0.21 | 0.19 |
|  | 0.15 | 0.43 | 0.33 | 0.31 | 0.42 |
|  | 1.47 | 2.58 | 2.30 | 2.56 | 4.07 |
|  | 0.46 | 0.40 | 0.81 | 0.77 | 0.74 |
|  | 0.71 | 1.39 | 0.96 | 1.44 | 1.11 |
|  | 2.32 | 2.35 | 3.85 | 4.14 | 2.33 |
| Mean (SEM) | 1.14 (0.39) | 1.42 (0.41) | 2.21 (0.93) | 2.00 (0.68) | 1.82 (0.62) |

**Fig 9 and S_Fig 4 p21**

Results of p21 in real time RT-PCR (n = 3) as fold change relative to untreated control calculated using the ΔΔCp-method and individual data points calculated using the ΔCp-method and densitometric protein results in Western blot (n = 3) as fold change and integrated signal intensity; normalised to the housekeeper α-actin in A549 and A549^r^CDDP^2000^ cells.

| Cell line | A549 | | A549^r^CDDP^2000^ | | |
| --- | --- | --- | --- | --- | --- |
| Treatment concentration | control | 11 µM | control | 11 µM | 34 µM |
| mRNA (fold change relative to untreated control) | | | | | |
|  |  | 9.496 |  | 2.534 | 8.619 |
|  |  | 6.239 |  | 2.611 | 5.199 |
|  |  | 6.713 |  | 1.921 | 5.206 |
| Mean (SEM) |  | 7.483 (1.016) |  | 2.355 (0.218) | 6.341 (1.139) |
| mRNA (individual data points) | | | | | |
|  | 0.013 | 0.162 | 0.025 | 0.070 | 0.271 |
|  | 0.023 | 0.174 | 0.033 | 0.096 | 0.205 |
|  | 0.0130 | 0.107 | 0.018 | 0.038 | 0.115 |
| Mean (SEM) | 0.016 (0.003) | 0.148 (0.021) | 0.026 (0.004) | 0.068 (0.017) | 0.197 (0.045) |
| Protein (fold change relative to untreated control) | | | | | |
|  |  | 1.73 |  | 1.16 | 1.76 |
|  |  | 1.56 |  | 0.89 | 1.04 |
|  |  | 1.29 |  | 1.18 | 1.15 |
| Mean (SEM) |  | 1.53 (0.13) |  | 1.08 (0.09) | 1.32 (0.22) |
| Protein (integrated signal intensity) | | | | | |
|  | 1.84 | 3.19 | 1.59 | 1.84 | 2.80 |
|  | 0.95 | 1.48 | 1.49 | 1.33 | 1.55 |
|  | 1.32 | 1.70 | 1.43 | 1.69 | 1.65 |
| Mean (SEM) | 1.37 (0.26) | 2.12 (0.54) | 1.50 (0.05) | 1.62 (0.15) | 2.00 (0.40) |

**Fig 10 and S_Fig 5 SIP**

Results of p21 in real time RT-PCR (n = 3) as fold change relative to untreated control using the ΔΔCp-method and individual data points calculated using the ΔCp-method and densitometric protein results in Western blot (n = 5) as fold change and integrated signal intensity; normalised to the housekeeper α-actin in A549 and A549^r^CDDP^2000^ cells.

| Cell line | | A549 | | | | A549^r^CDDP^2000^ | | | | |
| --- | --- | --- | --- | --- | --- | --- | --- | --- | --- | --- |
| Treatment concentration | | control | | 11 µM | | control | | 11 µM | 34 µM | |
| mRNA (fold change relative to untreated control) | | | | | | | | | | |
|  | |  | | 7.836 | |  | | 1.569 | 1.811 | |
|  | |  | | 5.633 | |  | | 1.229 | 1.171 | |
|  | |  | | 3.601 | |  | | 1.495 | 2.695 | |
| Mean (SEM) | |  | | 5.690 (1.223) | |  | | 1.431 (0.103) | 1.892 (0.442) | |
| mRNA (individual data points) | | | | | | | | | | |
|  | | 2.6*10^-4^ | 2.2*10^-3^ | | 7.8*10^-4^ | | 1.3*10^-3^ | | | 1.5*10^-3^ |
|  | | 5.0*10^-4^ | 3.0*10^-3^ | | 1.2*10^-3^ | | 1.5*10^-3^ | | | 1.4*10^-3^ |
|  | | 3.8*10^-4^ | 1.4*10^-3^ | | 6.0*10^-4^ | | 9.1*10^-4^ | | | 1.7*10^-3^ |
| Mean (SEM) | | 3.8*10^-4^ (7.0*10^-5^) | 2.2*10^-3^ (4.6*10^-4^) | | 8.5*10^-4^ (1.6*10^-4^) | | 1.2*10^-3^ (1.5*10^-4^) | | | 1.5*10^-3^ (9.3*10^-5^) |
| Protein (fold change relative to untreated control) | | | | | | | | | | |
|  | |  | | 1.40 | |  | | 0.87 | 0.84 | |
|  | |  | | 1.26 | |  | | 1.22 | 1.31 | |
|  | |  | | 0.58 | |  | | 1.31 | 1.35 | |
|  | |  | | 0.74 | |  | | 0.63 | 0.73 | |
|  | |  | | 0.84 | |  | | 0.73 | 0.75 | |
| Mean (SEM) | |  | | 0.96 (0.16) | |  | | 0.95 (0.13) | 0.99 (0.14) | |
| Protein (integrated signal intensity) | | | | | | | | | | |
|  | 0.47 | | | 0.65 | | 1.50 | | 1.30 | 1.25 | |
|  | 0.40 | | | 0.51 | | 1.12 | | 1.36 | 1.47 | |
|  | 0.59 | | | 0.34 | | 0.90 | | 1.18 | 1.22 | |
|  | 0.60 | | | 0.45 | | 1.79 | | 1.13 | 1.30 | |
|  | 0.47 | | | 0.39 | | 0.98 | | 0.72 | 0.73 | |
| Mean (SEM) | 0.51 (0.04) | | | 0.47 (0.05) | | 1.26 (0.17) | | 1.14 (0.11) | 1.20 (0.12) | |

**Fig 11 and S_Fig 6 XPC**

Results of XPC in real time RT-PCR (n = 3) as fold change relative to untreated control using the ΔΔCp-method and individual data points calculated using the ΔCp-method and densitometric protein results in Western blot (n = 5) as fold change and integrated signal intensity; normalised to the housekeeper α-actin in A549 and A549^r^CDDP^2000^ cells.

| Cell line | A549 | | A549^r^CDDP^2000^ | | |
| --- | --- | --- | --- | --- | --- |
| Treatment concentration | control | 11 µM | control | 11 µM | 34 µM |
| mRNA (fold change relative to untreated control) | | | | | |
|  |  | 3.385 |  | 1.572 | 1.586 |
|  |  | 2.783 |  | 1.005 | 0.849 |
|  |  | 2.505 |  | 1.414 | 2.210 |
| Mean (SEM) |  | 2.891 (0.260) |  | 1.331 (0.167) | 1.548 (0.393) |
| mRNA (individual data points) | | | | | |
|  | 0.008 | 0.032 | 0.021 | 0.034 | 0.034 |
|  | 0.009 | 0.030 | 0.023 | 0.023 | 0.019 |
|  | 0.007 | 0.020 | 0.011 | 0.016 | 0.026 |
| Mean (SEM) | 0.008 (0.001) | 0.027 (0.004) | 0.018 (0.004) | 0.024 (0.005) | 0.026 (0.004) |
| Protein (fold change relative to untreated control) | | | | | |
|  |  | 1.38 |  | 0.80 | 0.97 |
|  |  | 1.77 |  | 1.39 | 1.71 |
|  |  | 1.33 |  | 1.25 | 1.25 |
|  |  | 1.47 |  | 0.68 | 1.32 |
|  |  | 1.09 |  | 1.56 | 1.66 |
| Mean (SEM) |  | 1.41 (0.11) |  | 1.13 (0.17) | 1.38 (0.14) |
| Protein (integrated signal intensity) | | | | | |
|  | 0.93 | 1.28 | 1.21 | 0.97 | 1.18 |
|  | 0.83 | 1.48 | 0.96 | 1.33 | 1.64 |
|  | 0.93 | 1.23 | 1.07 | 1.34 | 1.34 |
|  | 0.72 | 1.06 | 1.15 | 0.78 | 1.52 |
|  | 1.20 | 1.31 | 1.33 | 2.08 | 2.22 |
| Mean (SEM) | 0.92 (0.08) | 1.27 (0.07) | 1.15 (0.06) | 1.30 (0.22) | 1.58 (0.18) |

**Fig 12 and S_Fig 7 GADD45a**

Results of GADD45a in real time RT-PCR (n = 3) as fold change relative to untreated control using the ΔΔCp-method and individual data points calculated using the ΔCp-method and densitometric protein results in Western blot (n = 4) as fold change and integrated signal intensity; normalised to the housekeeper α-actin in A549 and A549^r^CDDP^2000^ cells.

| Cell line | A549 | | A549^r^CDDP^2000^ | | |
| --- | --- | --- | --- | --- | --- |
| Treatment concentration | control | 11 µM | control | 11 µM | 34 µM |
| mRNA (fold change relative to untreated control) | | | | | |
|  |  | 5.497 |  | 1.719 | 2.727 |
|  |  | 3.014 |  | 1.667 | 1.770 |
|  |  | 3.036 |  | 1.306 | 2.088 |
| Mean (SEM) |  | 3.849 (0.824) |  | 1.564 (0.130) | 2.195 (0.282) |
| mRNA (individual data points) | | | | | |
|  | 0.005 | 0.034 | 0.018 | 0.034 | 0.058 |
|  | 0.010 | 0.037 | 0.024 | 0.043 | 0.046 |
|  | 0.007 | 0.027 | 0.012 | 0.017 | 0.029 |
| Mean (SEM) | 0.007 (0.002) | 0.033 (0.003) | 0.018 (0.003) | 0.031 (0.008) | 0.044 (0.008) |
| Protein (fold change relative to untreated control) | | | | | |
|  |  | 0.67 |  | 0.95 | 0.81 |
|  |  | 1.30 |  | 0.91 | 0.89 |
|  |  | 0.76 |  | 1.03 | 0.90 |
|  |  | 0.75 |  | 1.05 | 0.73 |
| Mean (SEM) |  | 0.87 (0.15) |  | 0.98 (0.03) | 0.83 (0.04) |
| Protein (integrated signal intensity) | | | | | |
|  | 1.15 | 0.77 | 1.51 | 1.44 | 1.22 |
|  | 0.81 | 1.05 | 0.86 | 0.77 | 0.76 |
|  | 0.78 | 0.59 | 0.87 | 0.89 | 0.78 |
|  | 0.94 | 0.70 | 1.22 | 1.28 | 0.88 |
| Mean (SEM) | 0.92 (0.09) | 0.78 (0.10) | 1.12 (0.16) | 1.10 (0.16) | 0.91 (0.11) |
